# A comparative meta-analysis of structural magnetic resonance imaging studies and gene expression profiles revealing the similarities and differences between late life depression and mild cognitive impairment

**DOI:** 10.1017/S0033291724002563

**Published:** 2024-11

**Authors:** Ling Zhao, Lijing Niu, Haowei Dai, Tatia M.C. Lee, Ruiwang Huang, Ruibin Zhang

**Affiliations:** 1 The Second School of Clinical Medicine, Southern Medical University, Guangzhou, PR China; 2 Cognitive Control and Brain Healthy Laboratory, Department of Psychology, School of Public Health, Southern Medical University, Guangzhou, PR China; 3 State Key Laboratory of Brain and Cognitive Sciences, The University of Hong Kong, Hong Kong, SAR China; 4 Laboratory of Neuropsychology and Human Neuroscience, The University of Hong Kong, Hong Kong, SAR China; 5 Guangdong-Hong Kong-Macao Greater Bay Area Center for Brain Science and Brain-Inspired Intelligence, Guangdong-Hong Kong Joint Laboratory for Psychiatric Disorders, Guangdong Basic Research Center of Excellence for Integrated Traditional and Western Medicine for Qingzhi Diseases, Guangzhou, China; 6 School of Psychology, South China Normal University, Guangzhou, China; 7 Department of Psychiatry, Zhujiang Hospital, Southern Medical University, Guangzhou, PR China

**Keywords:** gene expression profiling, late-life depression, meta-analysis, mild cognitive impairment, voxel-based morphometry

## Abstract

**Background:**

Late-life depression (LLD) predisposes individuals to cognitive decline, often leading to misdiagnoses as mild cognitive impairment (MCI). Voxel-based morphometry (VBM) can distinguish the profiles of these disorders according to gray matter (GM) volumes. We integrated findings from previous VBM studies for comparative analysis and extended the research into molecular profiles to facilitate inspection and intervention.

**Methods:**

We comprehensively searched PubMed and Web of Science for VBM studies that compared LLD and MCI cases with matched healthy controls (HCs) from inception to 31st December 2023. We included 13 studies on LLD (414 LLDs, 350 HCs) and 50 on MCI (1878 MCIs, 2046 HCs). Seed-based d Mapping with Permutation of Subject Images (SDM-PSI) was used for voxel-based meta-analysis to assess GM atrophy, spatially correlated with neuropsychological profiles. We then used multimodal and linear-model analysis to assess the similarities and differences in GM volumetric changing patterns. Partial least squares (PLS) regression and gene enrichment were employed for transcription-neuroimaging associations.

**Results:**

GM volumes in the left hippocampus and right parahippocampal gyrus are more affected in MCI, along with memory impairment. MCI was spatially correlated with a more extensive reduction in the levels of neurotransmitters and a severe downregulation of genes related to cellular potassium ion transport and metal ion transmembrane transporter activity.

**Conclusion:**

Compared to LLD, MCI exhibited more GM atrophy in the hippocampus and parahippocampal gyrus and lower gene expression of ion transmembrane transport. Our findings provided imaging-transcriptomic-genetic integrative profiles for differential diagnosis and precise intervention between LLD and MCI.

## Introduction

Late-life depression (LLD), depression diagnosed in older adults, affects approximately five million older adults over the age of 65 years (Zivin, Wharton, & Rostant, 2013). LLD is associated with severe health outcomes, including a high risk of mortality, cognitive deficits, and poor quality of life (Wei et al., 2019). Compared to regular major depressive disorder (MDD), cognitive deficits in LLD persist despite clinical recovery or psychopathological remission (Riddle et al., 2017); moreover, there is a decrease in the threshold of dementia due to underlying neurological and biomolecular abnormalities (Kuo, Lin, & Lane, 2021; Ly et al., 2021). Therefore, the timely and accurate diagnosis of LLD is important for public health.

LLD is often underdiagnosed or misdiagnosed as mild cognitive impairment (MCI) (Devita et al., 2022), a neurodegenerative disease characterized by memory impairment (Petersen & Negash, 2008) with a morbidity rate of over 15.56% in older adults (Bai et al., 2022). Both conditions commonly coexist (Ismail et al., 2017), LLD and MCI also share entwined neurodegenerative symptoms in cognition, such as memory impairment and execution deficits (Mukku et al., 2021; Panza et al., 2023). Population-based research has shown that both disorders are at least predisposing (Hu et al., 2020) and aggravating (Cooper, Sommerlad, Lyketsos, & Livingston, 2015) factors of each other. Some scientists even assume that there might be an LLD-MCI-dementia continuum in which depressive symptoms act as an early manifestation rather than a risk factor (Invernizzi, Simoes Loureiro, Kandana Arachchige, & Lefebvre, 2021). However, given the differences in prognosis and treatment (Association, 2013; Sperling et al., 2011), it is crucial to avoid misdiagnoses. The application of gray matter (GM) analysis (Minkova et al., 2017) combined with co-localized phenotypic traits, transcriptomic signatures, and genetic features are valuable tools for conceptualizing and studying the etiological basis of these disorders (Bao et al., 2023; Verheijen & Sleegers, 2018).

Previous studies have comprehensively enhanced our understandings of LLD and MCI separately, revealing GM volume reduction in frontostriatal-limbic regions in LLD (Agudelo, Aizenstein, Karp, & Reynolds, 2015) and the wide-spread GM atrophy in MCI involving the hippocampus and parahippocampal gyrus (Chen et al., 2020). Zackova, Jani, Brazdil, Nikolova, and Mareckova (2021) further explored the GM correlations between MDD and MCI, entailing shared volumetric reductions in the insula and superior temporal gyrus with other disease-specific structural changes. However, comparative studies between the two prevalent senile diseases are limited, hindering the clear distinction and early intervention in older adults. Additionally, to bridge the gap between structural findings and transcriptome (Fornito, Arnatkeviciute, & Fulcher, 2019), which could indicate potential diagnostic and therapeutic targets, it is necessary to conduct studies on neuroimaging-associated neurotransmitters (Aquilani et al., 2023; Jacobs, Baider, Goldzweig, Sapir, & Rottenberg, 2023) and gene expression (Cai et al., 2021; Liu, Abdellaoui, Verweij, & van Wingen, 2023), especially on neuronal processes such as synaptic transmission, anabolic, and biosynthetic pathways. Therefore, based on comparative changes in GM volume between MCI and LLD, we conducted further research into neuroimaging-associated neurotransmitters and transcriptomics.

To accurately and promptly differentiate between LLD and MCI in clinical practice, we performed a meta-analysis to examine the represented profiles in structure and gene expression. First, previous VBM studies were integrated for GM atrophy patterns and spatially correlated behavioral/disease profiles. Second, we identified the common and distinct GM volumetric changing patterns. Finally, based on the anatomical results, we decoded the associated neurotransmitter systems and gene expression profiles.

## Methods

### Literature search and selection

Our meta-analysis was conducted following the Preferred Reporting Items for Systematic Reviews and Meta-analyses (PRISMA) guidelines (Page et al., 2021). We systematically searched the PubMed and Web of Science databases from inception to 31st December 2023, to identify VBM studies on GM changes in LLD and MCI. The search strategy for LLD was (elder OR geriatric OR [late life] OR [late onset] OR older OR [old age]) AND depress* AND ([voxel-based morphometry] OR VBM). The search strategy for MCI was ([mild cognitive impairment] OR MCI) AND ([voxel-based morphometry] OR VBM).

The studies included in this meta-analysis were required for (i) original peer-reviewed studies; (ii) quantitative automated whole-brain GM assessment performed using VBM; (iii) comparison of the experimental group (i.e. MCI or LLD patients) to a matched healthy control (HC) group; (iv) provision of results as coordinates of activation foci in stereotactic space, either Montreal Neurological Institute (MNI) or Talairach reference space; and (v) selection of participants according to internationally recognized diagnostic criteria. In contrast, we excluded studies with (i) case reports; (ii) solely region-of-interest (ROI) analysis; (iii) only functional magnetic resonance imaging (fMRI), positron emission tomography (PET), electrophysiology; (iv) other psychiatric or neurological disorders, such as AD, vascular MCI (vMCI); (v) comorbidity with any other neurological or neurodegenerative diseases, such as Parkinson's disease (PD), multiple sclerosis (MS); and (vi) substantially overlapping patient populations with other studies. All the diagnoses of depression relied on DSM, and all the diagnoses of MCI also relied on DSM or reliable clinical criteria from Petersen et al. (2001) or the National Institute on Aging and Alzheimer's Association (NIA-AA). Detailed diagnostic tools and criteria are shown in online Supplementary Tables S1 and S2. In LLD studies, although ‘late-life’ is generally defined as 65 years or older, various LLD/MDD cut-offs (commonly ranging from 50 to 75 years) are currently acceptable in research (Baba et al., 2022). In MCI studies, a cut-off age is not part of the diagnostic criteria and therefore barely used in studies, but the overall prevalence has been estimated to be in the 12 to 18% range in persons over 60 years (Petersen, 2016). In our study, the mean age of LLDs was 69.97 and the mean age of MCIs was 71.57, which was acceptable for old age in literature on this topic.

To measure the quality of studies we included in the meta-analysis in consideration of reliability, we assessed the quality of each using the Newcastle-Ottawa scale (NOS) (Stang, 2010), included those with good (9–8) or moderate (7–5) quality, and excluded those with poor quality (4–0). In terms of comparability, we only included those studies with age-matched participants. The use of matched gender or education was not deemed necessary; however, we allotted one more score for matched studies. We did not consider handedness.

Initially, 775 MCI studies and 92 LLD studies were found during the systematic search. After carefully screening the studies according to their abstracts and full texts, we retained those studies that met the inclusion criteria. Among these, five MCI studies (Du et al., 2022; Gupta, Kim, Kim, & Kwon, 2020; Huang et al., 2023; van de Mortel, Thomas, van Wingen, & Alzheimer's Disease Neuroimaging, 2021; Xiong et al., 2023) obtained data from the Alzheimer's Disease Neuroimaging Initiative (ADNI) database (http://adni.loni.usc.edu) and two LLD studies (Harada et al., 2016, 2018) comprised participants that overlapped according to the author. We only retained studies with the largest group size for the meta-analysis. Regarding longitudinal studies, only data collected at baseline was utilized. We included and recorded studies that reported different subtypes, such as early/late MCI and AD converter/non-converter, as different datasets. We excluded the datasets of non-amnestic mild cognitive impairment (naMCI) when it was divided from amnestic mild cognitive impairment (aMCI) in consideration of consistency and specificity. This is because aMCI was closer to the earlier concept of MCI prior to updates (Petersen et al., 2014); moreover, it has more distinct structural and cognitive patterns compared to heterogeneous naMCI (Du, Dang, Chen, Chen, & Zhang, 2023; Qin et al., 2022), whose higher risk of progressing to AD makes it the focus in research concerning LLD-MCI-dementia continuum (Invernizzi et al., 2021).

**Figure 1.**
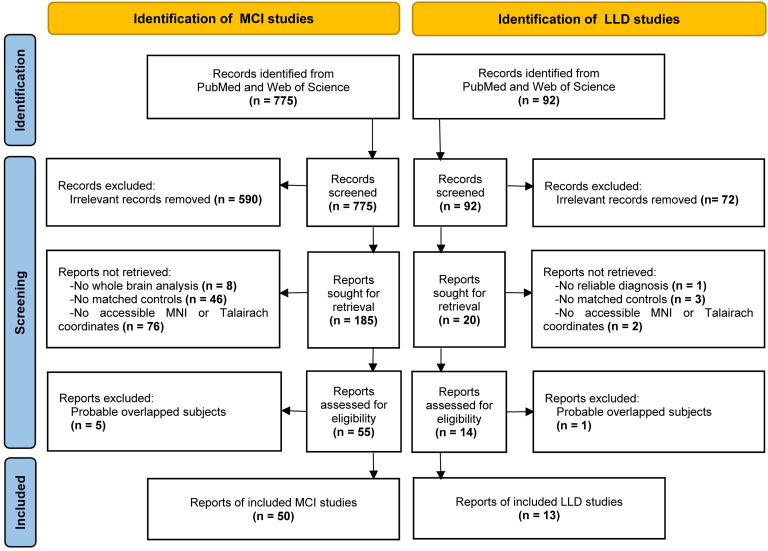
Flow diagram of inclusion and exclusion process of MCI (left) and LLD (right) studies.

Finally, we investigated 50 MCI studies with 55 datasets (1878 patients, 2046 HCs) and 13 LLD studies with 13 datasets (414 patients, 350 HCs). All of them were eligible for meta-analysis (NOS ⩾ 5), in which ten MCI datasets and two LLD datasets were classified as having good quality (NOS ⩾ 8) according to NOS (online Supplementary Tables S1 and S2). The detailed process of inclusion and exclusion is shown in the flow diagram in Fig. 1.

### Coordinate-based analysis

A voxel-based meta-analysis of neuroimaging studies was conducted using seed-based d Mapping (formerly Signed Differential Mapping) with Permutation of Subject Images (SDM-PSI) software for Windows 64 bits (www.sdmproject.com/). The voxel-based meta-analytic methods benefit from a more exhaustive and unbiased inclusion of studies (Muller et al., 2018; Radua & Mataix-Cols, 2012). SDM-PSI has been proven to be a conservative method that increases the statistical power when there are multiple effects, and is distinct from other current meta-analyses methods due to the use of effect sizes, random-effects models, Freedman-Lane-based permutations, and threshold-free cluster enhancement (TFCE) statistics (Albajes-Eizagirre, Solanes, Vieta, & Radua, 2019). To optimally balance sensitivity and specificity (Albajes-Eizagirre et al., 2019), the parameters were set using the modality of VBM-GM (anisotropy = 1, isotropic FWHM = 20 mm, mask = gray matter, voxel = 2 mm) and the default SDM threshold (*p* < 0.05 with peak *Z* > 1 and a cluster extent >10 voxels). To validate the significant clusters, we derived heterogeneity analysis using *I^2^* statistics and assessed publication bias by conducting Egger's test and generating funnel plots. *I^2^* < 50% was considered low heterogeneity (Higgins, Thompson, Deeks, & Altman, 2003). Egger-*p* > 0.05 and visually symmetric funnel plots indicate non-significant publication bias.

We recorded each article's peak coordinates and corresponding statistics (*t*, *z,* or *p* values), with their software packages, stereotactic spaces, and threshold according to the SDM tutorial. First, we separately investigated the GM alteration within each group (LLD *v.* HC or MCI *v.* HC). Second, we detected regions with common GM atrophy by conducting Multimodal analysis (Radua, Romeo, Mataix-Cols, & Fusar-Poli, 2013) and compared the discrepant GM atrophy by conducting Linear model analysis (Radua, van den Heuvel, Surguladze, & Mataix-Cols, 2010). Third, the *p* values maps were used for further investigation.

### Behavioral and disease decoding

Data taken from the BrainMap database (http://www.brainmap.org/), which documents over 20 years of published functional brain imaging studies, was used to separately decode the structural changes in behavior and disease. Behavioral profiles span action, cognition, emotion, interoception, and perception, while disease profiles were generalized from neurological disorders according to the International Classification of Diseases (ICD). Multi-image Analysis GUI (Mango) (https://mangoviewer.com) was used to compute and compare the fraction of coordinates falling within the ROI with the fraction uniformly distributed (Lancaster et al., 2010, 2011). Behavioral or disease association is indicated when the difference between these fractions is significant (*Z* score ⩾ 3.0), viz Bonferroni corrected to an overall *p* value of 0.05 for all behavior sub-domains (Lancaster et al., 2012).

### Spatial correlation of receptor/transporter

JuSpace (https://github.com/juryxy/JuSpace) was used to quantify the co-localization between disease and altered expression of respective neurotransmitter systems based on the topographical relationship. Unthresholded meta-analysis maps testing for regions showing and those not showing convergence in the meta-analyses were used for Pearman's rank partial correlation analysis with default settings (controlling for partial volume effects and spatial autocorrelation using underlying gray matter probability) (Dukart et al., 2021). We included receptor maps covering cholinergic, dopaminergic, GABAergic, glutamatergic, noradrenergic, opioidergic, and serotonergic neurotransmission. Receptor maps showing a significant association with respective brain maps of reduced GM volume entered multiple linear regression analyses to test for specificity. Statistical significance was set at *p* < 0.05 (FWE rate corrected) to control the rate of false positives in multiple comparisons (Glickman, Rao, & Schultz, 2014).

### Transcription-neuroimaging association

The microarray-based gene expression data were acquired from the Allen Human Brain Atlas (AHBA) database (Hawrylycz et al., 2015). The gene expression data and *Z*-map of LLD and MCI were preprocessed through a recommended pipeline (Arnatkeviciute, Fulcher, & Fornito, 2019; Glasser et al., 2016), with further details provided in the online Supplementary Methods.

Partial least squares (PLS) regression (Abdi & Williams, 2013) was employed to identify the transcriptional profiles associated with abnormal GM volume in LLD and MCI. The PLS components were ranked based on the variance explained between the independent variable (gene expression matrix) and dependent variable (case–control *t* vector). A spatial autocorrelation (SA) corrected permutation test was adopted to examine whether the *R^2^* of the PLS component was significantly greater than that expected by chance. For each significant component, the bootstrapping method was used to correct estimation errors and rank the contribution of the weight of each gene. We ranked these genes descending by their corrected weight in significant PLS components. GOrilla (http://cbl-gorilla.cs.technion.ac.il/) was used for gene enrichment analysis, which identified enriched GeneOntology (GO) terms (Eden, Navon, Steinfeld, Lipson, & Yakhini, 2009). All ontology categories were considered, including biological process, molecular function, and cellular components. Significant enrichment was set to Benjamini-Hochberg false discovery rate (FDR)-corrected *q* < 0.05 (Xia et al., 2022).

## Results

### Decoding GM alteration in LLD

**Figure 2.**
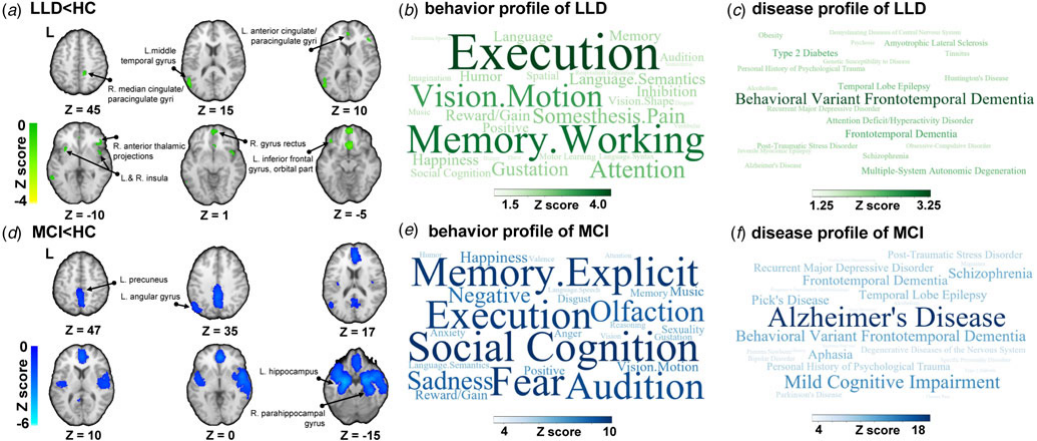
Regions with GM atrophy in LLD/MCI and their related behavioral/disease profiles. (a) Regions of GM volume decreases in LLDs compared to HCs; (b) Behavior profile of LLD related to GM atrophy; (c) Disease profile of LLD related to GM atrophy; (d) Regions of GM volume decreases in MCIs compared to HCs; (e) Behavior profile of MCI related to GM atrophy; (f) disease profile of MCI related to GM atrophy. (GM, gray matter; LLD, late-life depression; MCI, mild cognitive impairment; HC, healthy controls; Voxel-wise threshold *p* < 0.05 uncorrected; minimum cluster extent 10 voxels.).

We investigated 13 datasets on LLD (414 patients, 350 HCs). Basic information, diagnostic tools and quality assessment for each study, along with detailed demographic and clinical characteristics, are shown in online Supplementary Table S1. We gained a holistically balanced demographic profile by including only studies with participants of similar ages, alongside accounting for gender and education when evaluating study quality, which facilitates a comprehensive and credible neurobiological insight across the entire elderly population. Compared to HCs, patients with LLD showed a decrease in GM volume in the right gyrus rectus, left middle temporal gyrus, left inferior frontal gyrus (orbital part), right anterior thalamic projections, left anterior cingulate/paracingulate gyri, right insula, right median cingulate/paracingulate gyri, left insula, and left middle temporal gyrus (Fig. 2a, online Supplementary Table S3). No regions with increased GM volumes were observed. Heterogeneity analysis using *I*^2^ statistics (1.60–35.04%) showed no significant variability between studies, and quantitative assessment of Egger's test (*p* = 0.339–0.928) showed no publication bias in all significant brain regions.

Regarding behavioral analysis, the obtained meta-analysis map of LLD corresponds to executive function (*Z* = 4.44), working memory (*Z* = 3.80), and motion vision (visual perception that receives motor-related input) (*Z* = 3.03) (Fig. 2b). Regarding disease analysis, we found behavioral variant frontotemporal dementia (bvFTD) (*Z* = 3.30) to be the most relevant disease (Fig. 2c).

### Decoding GM alteration in MCI

We investigated 55 datasets on MCI (1878 patients, 2046 HCs). Detailed information, diagnostic tools and quality assessment for each study, as well as the summarized demographic and clinical characteristics are shown in online Supplementary Table S2 in the Supplementary. The well-matched qualified literature included allowed for a more comprehensive and credible neurobiological evaluation whithin the population. We noticed that, compared to HCs, patients with MCI showed decreased GM volume in the right parahippocampal gyrus, left hippocampus, left precuneus, and left angular gyrus (Fig. 2d, online Supplementary Table S3). No region with an increased GM volume was observed. Heterogeneity analysis using *I*^2^ statistics showed no variability in the regions of the left hippocampus (5.96%) and left precuneus (0.21%) between the studies, while there was variability in the regions of the right parahippocampal gyrus and left angular gyrus. A quantitative assessment of Egger's test (*p* = 0.181–0.967) showed no publication bias in all significant brain regions.

Behavioral analysis (Fig. 2e) revealed that the obtained meta-analysis map of MCI significantly correspond to functions in varied domains, including social cognition (*Z* = 10.14), fear (*Z* = 9.36), explicit memory (*Z* = 9.83), and executive function (*Z* = 9.63). Regarding disease analysis (Fig. 2f), a large number of diseases were found to be related, including AD (*Z* = 19.90), MCI (*Z* = 13.28), bvFTD (*Z* = 10.881), schizophrenia (*Z* = 10.113), and frontotemporal Dementia (*Z* = 9.629).

### Comparison of GM atrophy and spatially correlated receptor/transporter densities

**Figure 3.**
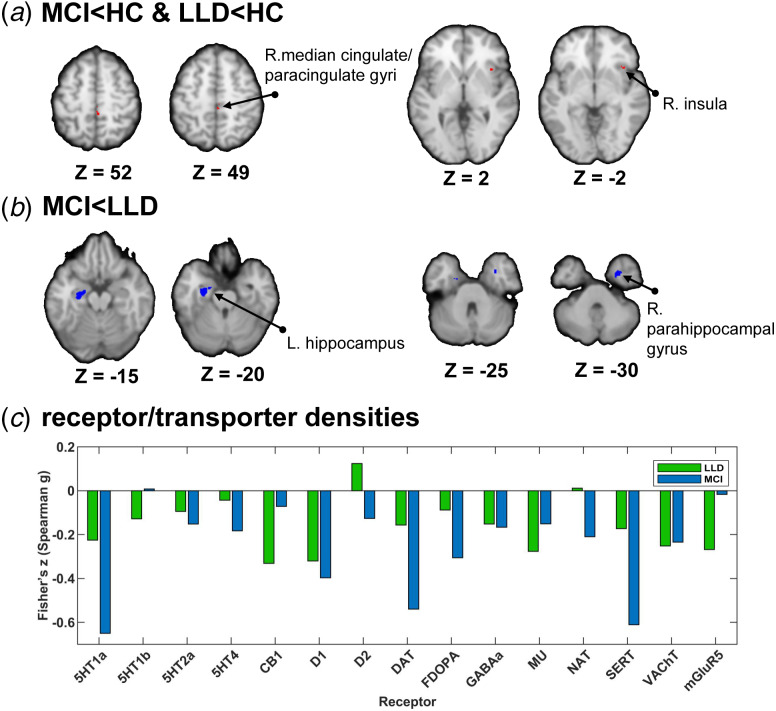
Comparison of GM-atrophic regions in LLD and MCI and their spatial-correlated neurotransmitter densities. (a) Shared regions with GM volume decrease in LLD and MCI; (b) Specific regions with GM atrophy in MCI in preference to LLD; (c) Receptor/transporter densities colocalized with different GM-atrophic regions in LLD and MCI. (GM, gray matter volume; LLD, late-life depression; MCI, mild cognitive impairment; HC, healthy controls; Voxel-wise threshold *p* < 0.05 uncorrected; minimum cluster extent 10 voxels; *p* < 0.0025 for conjunction meta-analysis; * *p* < 0.05).

Conjunction analyses showed that the right median cingulate/paracingulate gyri and the right insula exhibited significant reduced GM volume in both LLD and MCI (Fig. 3a, online Supplementary Table S4). We separately tested the heterogeneity of the regions above in the LLD *v.* HCs group and the MCI *v.* HCs group. No significant corresponding features were found in behavioral/disease analysis (*Z* < 3). Regression analysis was used to compare LLDs and MCIs, where MCI showed more significant reductions in GM volume in the left hippocampus and right parahippocampal gyrus (Fig. 3b, online Supplementary Table S5). We found that atrophic regions specific to MCI corresponded to explicit memory (*Z* = 3.45) in behavioral analysis and were related to AD (*Z* = 4.30) in disease analysis.

JuSpace was used to link the neuroimage to neurotransmitter information, which included receptor/transporter maps covering cholinergic, dopaminergic, GABAergic, glutamatergic, noradrenergic, opioidergic, and serotonergic neurotransmission (Fig. 3c). In LLD, the neurotransmitters that were significantly correlated were CB1 (Fisher'*z* = −0.331, *p* = 0.023) at opioidergic synapses, D1 (Fisher'*z* = −0.320, *p* < 0.001) at dopaminergic synapses, VAChT (Fisher'*z* = −0.252, *p* = 0.007) at cholinergic synapses, and mGluR5 (Fisher'*z* = −0.268, *p* = 0.043) at glutamatergic synapses. In MCI, we found that GM morphological abnormalities were significantly correlated within 5-HT1A (Fisher'*z* = −0.650, *p* < 0.001), 5-HT4 (Fisher'*z* = −0.182, *p* = 0.049) and SERT (Fisher'*z* = −0.611, *p* = 0.001) at serotonergic synapses; D1 (Fisher'*z* = −0.397, *p* < 0.001), DAT (Fisher'*z* = −0.540, *p* < 0.001), and FDOPA (Fisher'*z* = −0.306, *p* = 0.001) at dopaminergic synapses; NAT (Fisher'*z* = −0.210, *p* = 0.022) at noradrenergic synapses; and VAChT (Fisher'*z* = −0.235, *p* = 0.013) at cholinergic synapses.

### Gene expression profiles related to GM volume alteration in MCI

**Figure 4.**
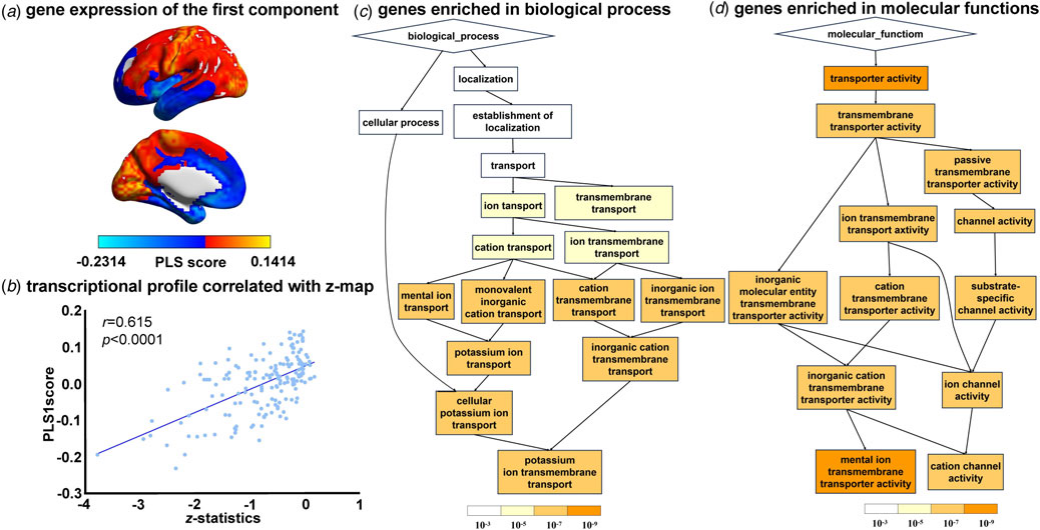
Association between gray matter volume alternation in MCI and gene expressions. (a) A gene expression profile identified by the first PLS component; (b) The transcriptional profiles were positively correlated with the *z-*map of the gray matter volume differences; (c) Genes ranked in ascending order of the PLS 1 weight were enriched in the biological process of cellular potassium ion transport (FDR-corrected *q* < 0.05); (d) The molecular function of metal ion transmembrane transporter activity (FDR-corrected *q* < 0.05).

We obtained normalized gene expression data of 10 027 genes for 176 ROIs of HCP atlas from the AHBA data and set these expression data as the predictor variables in PLS. The *Z*-maps, depicting the differences in GM volume among individuals diagnosed with MCI/LLD and healthy controls across the 176 ROIs based on the HCP atlas, were employed as the dependent variable in the PLS. Only the first component of the PLS regression with MCI *Z*-map was significant and explained 36.17% of the variance in the MCI-related alteration in GM volume (*p* < 0.05 for component 1, permutation tests with spatial autocorrelation corrected). The first component represented a transcriptional profile characterized by high expression, mainly in the left anterior agranular insula complex in the HCP atlas (Fig. 4a), a cytoarchitecturally distinct sub-region in the insula. The *Z*-map of gray matter volume difference between MCI and healthy controls was significantly positive with the regional mapping of the first component (*r* = 0.6015, *p* < 0.0001, Fig. 4b). Results from the Gene Ontology enrichment analysis revealed that the genes ranked in descending order of the first component weight were enriched in biological processes including cellular potassium ion transport, molecular function related to metal ion transmembrane transporter activity (Fig. 4c, 4d, FDR-corrected *q* < 0.05). No significant enrichment of cellular components was observed.

## Discussion

To facilitate timely differentiation between LLD and MCI, we conducted a meta-analysis examining associated profiles of atrophic structures alongside decoding of neuropsychological features, neurotransmission and gene expression. We observed reduced GM volumes in the right median cingulate/paracingulate gyri and the right insula in both conditions, whereas the left hippocampus and the right parahippocampal gyrus demonstrated more pronounced reductions specifically in MCI. This indicates that executive function is essential in these two diseases, while memory deficit is more relevant in aMCI. In terms of the neurotransmission system, D1 at dopaminergic synapses and VAChT at cholinergic synapses play prominent roles in LLD, while MCI is related to serotonergic synapses including 5-HT1A and SERT, dopaminergic synapses including D1, DAT, and FDOPA, together with various other neurotransmitters such as cholinergic synapses. In the assessment of gene expression, the first component was significant in MCI, which was highly expressed in the left anterior insula complex in the HCP atlas and enriched in processes including cellular potassium ion transport and metal ion transmembrane transporter activity.

### GM morphological abnormalities differ between LLD and MCI

GM reduction in the left hippocampus and the right parahippocampal gyrus was more specific in MCIs than in LLDs, which correspond to explicit memory and AD. The hippocampus, whose subfields selectively correspond to different cognitive domains (Liu, Liu, Qiu, & Alzheimer's Disease Neuroimaging, 2021), is also vulnerable in deleterious conditions (Zhang et al., 2022), and shows structural and biochemical changes when MCI occurs or progresses (Geerlings & Gerritsen, 2017; Wang et al., 2024). Specifically, reducing cornu ammonis 1 (CA1) in the hippocampus is a pathway that links LLD to persistent cognitive decline, such as aMCI and AD (Choi et al., 2017). The hippocampus also collaborates with other cortico-subcortical structures such as the parahippocampal gyrus (PHG), integrating into the Papez circuit and participating in high-level cognitive processes such as episodic memory synchronization (Forno, Llado, & Hornberger, 2021). PHG is proven to have significant volume differences when comparing MCI to HCs and ADs to MCIs (Echavarri et al., 2011), due to neuronal degeneration (Wang et al., 2017), and abnormalities in microvasculature-associated gene expression (Katsel et al., 2018). The observed variability in our findings in MCI could be attributable to its variability in neuropsychiatric symptoms. The structural involvement of the fronto-limbic circuit corresponds to specific neuropsychiatric symptoms (Cotta Ramusino et al., 2024). For instance, researchers have related the right parahippocampal gyrus to cognitive reserve (Zhou et al., 2024) and the left angular gyrus to financial capacity (Nowrangi et al., 2022) in MCI. Potential confounding factors, such as age (Taylor et al., 2020) or medication (Chan, Yiu, Kwok, Wong, & Tsoi, 2019), might also explain these findings; however, subgroup analysis was not conducted due to the relatively uniform demographics and finite number of studies.

### GM morphological abnormalities have common ground between LLD and MCI

The right insula and right median cingulate/paracingulate gyri are the two shared reduced regions in LLD and MCI. Both regions have direct structural connections with various systems involved in these two diseases. The insula serves a wide variety of functions in concert within and across functional networks, ranging from sensory and motor functions to high-level cognition such as primordial and social emotions (Fermin et al., 2023; Uddin, Nomi, Hebert-Seropian, Ghaziri, & Boucher, 2017). This is consistent with the reports of previous studies, as Zackova et al. (2021) suggested that the decreased volume of the insula may reflect communication deficits and may be regarded as a risk factor for both MDD and MCI because of the consequent infrequent participation in mental or social stimulating activities. Moreover, the median cingulate motor area is allowed to output action-outcome learning to premotor areas after the posterior cingulate cortex receives spatial and action-related information from the hippocampal system and parietal cortical areas (Rolls, 2019), which is part of the Papez circuit that is related to both emotions and memory (Forno et al., 2021).

### Discriminating patterns of spatial correlated neurotransmission and transcription

Dopamine (DA) plays significant roles in numerous cognitive functions, including memory processing and behavioral formations (Chen, Chen, Kim, & Xiong, 2021). The cholinergic system, including acetylcholine (ACh) and vesicular acetylcholine transporter (VAChT), acts as a modulator of cognition in terms of attention, learning, reward behavior, and memory (Hampel et al., 2018; Han et al., 2017). Moreover, cholinergic innervation interacts with DA (Skirzewski et al., 2022). The impact of serotonergics is noticeable in MCI (Smith et al., 2017), in accordance with novel therapeutics in AD containing psychedelics (Garcia-Romeu, Darcy, Jackson, White, & Rosenberg, 2022). Furthermore, the significant effects of opioidergic (Browne, Jacobson, & Lucki, 2020) and glutamatergic synapses (Lissemore et al., 2018) in LLD are consistent with those reported by research for novel targets.

Functional enrichment analysis of significant genes has revealed a functional imbalance of neurotransmitters and synapses in AD (Scaduto et al., 2023). The anterior agranular insula complex is an essential node in projections connected to the hippocampus (Cenquizca & Swanson, 2007), the central medial nucleus, and the striatal and limbic forebrain circuitry (Vertes, Hoover, & Rodriguez, 2012). Ion channels not only maintain water/ion metabolism homeostasis but also moderate the signaling pathways of neurons and glial cells, and their dysfunction are significant pathological features and new therapeutic targets for neurodegenerative disorders (Wang et al., 2022). For example, depressed Na^+^/K^+^ ATPase levels and impaired glutamate clearance in AD brain could lead to a cellular ion imbalance and electrophysiological dysfunction, probably triggered by amyloid beta peptide (A*β*) (Vitvitsky, Garg, Keep, Albin, & Banerjee, 2012). Additionally, dysregulation of neuronal iron homeostasis is likely to be an alternative unifying effect of early-onset familial AD (Lumsden et al., 2018).

### Strength and limitations

This study has some limitations. First, the amount of LLD research was finite, which might influence the representativeness and repeatability of the results. Future research should aim to include more studies on LLD to validate these findings. Second, there was some variability in the clinical implementation of LLD and MCI, which was mainly caused by disparities in the chosen cut-off values (Baba et al., 2022) and inner heterogeneity (Cotta Ramusino et al., 2024). Either LLD or MCI may be comparatively broad concepts, supplemented by conceptions including late-onset depression (LOD) (Olgiati, Fanelli, & Serretti, 2023), aMCI, and naMCI (Csukly et al., 2016). However, a more in-depth study could not be conducted due to the limited amount of available data. Future research should aim to standardize the identification and inclusion of subgroups to validate these findings. Third, the influence of medications was not thoroughly addressed in the analysis, and this could be a potential confounding factor in the interpretation of the results (Chan et al., 2019). Only 14 out of 55 datasets on MCI and 9 out of 13 datasets on LLD reported medication use, whose reported usage varied widely from 0% to 100% and differed in terms of treatment, making it impractical to separate our analysis based on medication use. Hence, the elaboration of medication use in future studies is encouraged.

Despite these limitations, we conducted a systematic literature search and included several datasets to conduct a comprehensive analysis of the commonalities and discrepancies between LLD and MCI. We chose parameters and thresholds for each research method meticulously adhering to the established manuals, which ensured and maximized the reliability of each part of the study, and balanced the sensitivity and specificity. Moreover, integrating neuroimaging findings with spatially correlated neurotransmitter data and gene expression profiles facilitated a better understanding of the structural and molecular differences between LLD and MCI. Finally, GM atrophy in the hippocampus and parahippocampal gyrus, alongside lower gene expression in ion transmembrane transport, can be used to accurately differentiate MCI from LLD. Prior studies have highlighted the potential of morphometric and molecular profiles as biomarkers due to their early manifestation prior to relatively overlapping and intricate symptomatic patterns (Cai et al., 2021; Liu et al., 2023). These features also serve as promising targets for timely and accurate therapeutic approaches, because rebalanced or imbalanced gene expression and synaptic transmission are believed to underpin changes in brain structure and clinical manifestation (Aquilani et al., 2023; Jacobs et al., 2023).

Overall, our study provides valuable insights into the structural and molecular profiles of LLD and MCI. Future research should address the abovementioned limitations and further explore the impact of clinical factors and treatment interventions on neuroimaging and molecular findings. Additionally, the clinical implications of the study's findings should be validated through prospective studies and clinical trials to confirm their utility in the differential diagnosis and management of LLD and MCI.

## Conclusions

In conclusion, GM volume in the left hippocampus and right parahippocampal gyrus is more likely to be affected in MCI along with memory impairment, while GM atrophy in the right insula and right median cingulate/paracingulate gyri coexist in both LLD and MCI. MCI is related to extended downregulation in various neurotransmissions besides dopaminergic and cholinergic synapses, coupling with downregulation of genes enriched in ion transmembrane transporter activity. We infer that these imaging-transcriptomic-genetic integrative profiles can serve as potential targets for timely differential diagnosis and precise intervention between LLD and MCI.

## Supporting information

Zhao et al. supplementary material Zhao et al. supplementary material

## Supplementary material

The supplementary material for this article can be found at https://doi.org/10.1017/S0033291724002563
