# Supplementary material for: A comparative meta-analysis of structural magnetic resonance imaging studies and gene expression profiles revealing the similarities and differences between late life depression and mild cognitive impairment
Source: Psychol Med. 2024 Nov 25;54(15):4264–73. doi: 10.1017/S0033291724002563 (PMC11650184; doi:10.1017/S0033291724002563)
Supplement: Zhao et al. supplementary material [file S0033291724002563sup001.docx]

**Supplementary**

A comparative meta-analysis of structural magnetic resonance imaging studies and gene expression profiles revealing the similarities and differences between late life depression and mild cognitive impairment

Zhao Ling^1+^, Niu Lijing^2+^, Dai Haowei^2+^, Lee M.C. Tatia^3,4,5^, Ruiwang Huang^6^, Zhang Ruibin^2, 7,5^

1. The Second School of Clinical Medicine, Southern Medical University, Guangzhou, PR China
2. Cognitive Control and Brain Healthy Laboratory, Department of Psychology, School of Public Health, Southern Medical University, Guangzhou, PR China
3. State Key Laboratory of Brain and Cognitive Sciences, The University of Hong Kong, Hong Kong, SAR China
4. Laboratory of Neuropsychology and Human Neuroscience, The University of Hong Kong, Hong Kong, SAR China
5. Center for Brain Science and Brain-Inspired Intelligence, Guangdong-Hong Kong-Macao Greater Bay Area
6. School of Psychology, South China Normal University, Guangzhou, China.
7. Department of Psychiatry, Zhujiang Hospital, Southern Medical University, Guangzhou, PR China

+These authors contributed equally to this work.

Correspondence and request for materials should be addressed to:

Ruibin Zhang, Cognitive Control and Brain Healthy Laboratory, Department of Psychology, School of Public Health, Southern Medical University, E-mail: ruibinzhang@foxmail.com

**Catalogue**

[Preprocessing of gene expression from the Allen Human Brain Atlas (AHBA) database and Z-map of LLD and MCI 1](#_Toc1034)

*[Table S1.](#_Toc8352)* [Demographic, clinical characteristics, and quality assessment of LLD studies. 2](#_Toc8352)

*[Table S2.](#_Toc29993)* [Demographic, clinical characteristics, and quality assessment of MCI studies. 4](#_Toc29993)

*[Table S3.](#_Toc27104)* [Results of meta-analysis, heterogeneity assessment and publication bias of GM atrophy in LLD and MCI. 9](#_Toc27104)

*[Table S4.](#_Toc8504)* [Shared abnormal regions in GM between LLD and MCI. 10](#_Toc8504)

*[Figure S1.](#_Toc13667)* [Funnel plot analysis for significant coordinates in comparison between LLDs and HCs. 12](#_Toc13667)

*[Figure S2.](#_Toc6713)* [Funnel plot analysis for significant coordinates in comparison between MCIs and HCs. 13](#_Toc6713)

*[Figure S3.](#_Toc22501)* [Funnel plot analysis for significant coordinates in conjunction analysis (shared abnormality between LLD and MCI). 14](#_Toc22501)

[Included studies on late life depression 15](#_Toc10697)

[Included studies on mild cognitive impairment 16](#_Toc25876)

[References 20](#_Toc27455)

**Preprocessing of gene expression from the Allen Human Brain Atlas** **(AHBA) database and Z-map of LLD and MCI**

The microarray-based gene expression data were acquired from the Allen Human Brain Atlas (AHBA) website (<https://human.brain-map.org/>). The gene expression datasets were extracted from the post-mortem brain tissues of six donors, who were a 24-year-old (H0351.2001) and a 39-year-old (H0351.2002) African American male; a 57-year-old (H0351.1009), a 31-year-old (H0351.1012) and a 55-year-old (H0351.1016) European-ancestry male; as well as a 49-year-old Hispanic female (H0351.1015) (Hawrylycz et al., 2015). The profiles included 20,737 gene expressions represented by 58,692 probes. As right hemisphere samples were only available in two out of the six donors in the AHBA dataset, only tissue samples from the left hemisphere (n = all 6 donors) were included.

The expression data were preprocessed according to the 5 major steps: (i) probe-to-gene annotations; (ii) data filtering to discriminate expression signal from noise; (iii) probe selection to measure the expression level of a single gene at different exon; (iv) assigning and mapping samples to the HCP atlas (https://humanconnectome.org/); (v) normalizing expression values to account for outlying values and inter-participant variances. The open-source code concerning the preprocessing procedure was used (<https://github.com/BMHLab/AHBAprocessing>). After preprocessing, each tissue sample had 10,027 probes. All brain tissue samples were then spatially coregistered to a cortical parcellation atlas with 360 regions from the human connectome project (HCP) (Glasser et al., 2016). The mean gene expression value of all brain tissue samples in each region was calculated, and the expression level of all genes in the left hemisphere regions was used for regression analysis.

***Table S1.* Demographic, clinical characteristics, and quality assessment of LLD studies.**

| study | Diagnostic  Tool | Sample Size  (Female%) | | Age  mean (SD) | | Education  mean (SD) | | MMSE  mean (SD) | | Medication | NOS |
| --- | --- | --- | --- | --- | --- | --- | --- | --- | --- | --- | --- |
|  |  | LLD | HCs | LLD | HCs | LLD | HCs | LLD | HCs |  |  |
| Colloby et al. ([2011](file://C:\\Users\\Administrator\\Desktop\\Supp_20240207.docx#_ENREF_1)) | DSM-IV | 38  (71%) | 30  (67%) | 74.10  (6.10) | 74.40  (6.40) | - | - | 28.80  (1.10) | 29.50  (0.80) | - | 6 |
| Egger et al. ([2008](file://C:\\Users\\Administrator\\Desktop\\Supp_20240207.docx#_ENREF_2)) | DSM-IV | 14  (71%) | 20  (65%) | 71.40  (7.49) | 72.30  (7.70) | 9.40  (2.24) | 10.80  (2.69) | 27.21  (0.97) | 28.57  (0.81) | - | 8 |
| Harada et al. ([2016](file://C:\\Users\\Administrator\\Desktop\\Supp_20240207.docx#_ENREF_4)) | DSM-IV | 45  (58%) | 61  (72%) | 60.20  (8.20) | 62.90  (7.60) | 13.30  (2.30) | 13.60  (2.30) | 28.00  (2.20) | 28.80  (1.70) | 91% | 7 |
| Hwang et al. ([2010](file://C:\\Users\\Administrator\\Desktop\\Supp_20240207.docx#_ENREF_6)) | DSM-IV | 70  (0%) | 26  (0%) | 79.40  (5.30) | 79.50  (4.30) | 7.80  (4.60) | 8.10  (5.40) | 26.50  (3.40) | 27.80  (2.10) | - | 7 |
| Koolschijn et al. ([2010](file://C:\\Users\\Administrator\\Desktop\\Supp_20240207.docx#_ENREF_7)) | DSM-IV | 28  (100%) | 38  (100%) | 64.04  (10.90) | 61.89  (11.03) | 10.89  (4.05) | 11.08  (2.93) | 27.39  (2.46) | 28.54  (1.56) | 79% | 6 |
| Marano et al. (2015) | DSM-IV | 17  (59%) | 17  (47%) | 66.90  (6.40) | 66.00  (7.90) | 15.10  (2.40) | 15.40  (2.80) | 28.80  (0.70) | 28.20  (1.90) | 100% | 7 |
| Oudega et al. ([2014](file://C:\\Users\\Administrator\\Desktop\\Supp_20240207.docx#_ENREF_9)) | DSM-IV | 55  (66%) | 23  (52%) | 72.30  (7.80) | 70.30  (6.30) | - | - | - | - | 13% | 6 |
| Ribeiz et al. ([2013](file://C:\\Users\\Administrator\\Desktop\\Supp_20240207.docx#_ENREF_10)) | DSM-IV | 30  (77%) | 22  (77%) | 70.73  (6.59) | 70.41  (7.58) | 6.50  (5.49) | 9.91  (5.12) | 24.90  (4.19) | 27.95  (1.84) | 13% | 6 |
| Ries et al. ([2009](file://C:\\Users\\Administrator\\Desktop\\Supp_20240207.docx#_ENREF_11)) | DSM-IV | 15  (67%) | 32  (56%) | 66.30  (5.30) | 68.40  (7.40) | 14.60  (2.40) | 17.30  (2.30) | 29.00  (1.00) | 29.20  (1.90) | - | 7 |
| Sin et al. ([2018](file://C:\\Users\\Administrator\\Desktop\\Supp_20240207.docx#_ENREF_12)) | DSM-V | 29  (66%) | 23  (64%) | 68.58  (5.09) | 67.10  (4.80) | - | - | - | - | 100% | 5 |
| Tsa et al. ([2022](file://C:\\Users\\Administrator\\Desktop\\Supp_20240207.docx#_ENREF_13)) | DSM-IV | 36  (78%) | 17  (53%) | 65.60  (7.30) | 64.10  (7.90) | 9.70  (4.80) | 10.90  (5.20) | - | - | 89% | 6 |
| Xie et al. ([2012](file://C:\\Users\\Administrator\\Desktop\\Supp_20240207.docx#_ENREF_14)) | DSM-IV | 18  (48%) | 25  (48%) | 68.61  (6.81) | 74.28  (8.25) | 14.61  (2.57) | 15.32  (2.87) | 28.06  (1.21) | 28.92  (1.22) | 89% | 5 |
| Yuan et al. ([2008](file://C:\\Users\\Administrator\\Desktop\\Supp_20240207.docx#_ENREF_15)) | DSM-IV | 19  (53%) | 16  (50%) | 67.10  (7.20) | 67.70  (3.80) | 12.60  (4.30) | 13.10  (4.90) | 27.90  (2.60) | 28.10  (1.40) | 100% | 8 |
| Mean/Summary |  | 414  (57%) | 350  (61%) | 69.97 | 68.52 | 10.63 | 12.73 | 27.43 | 28.65 |  |  |

LLD, late-life depression; DSM: Diagnostic and Statistical Manual of Mental Disorders; MMSE, Mini Mental State Examination; NOS, Newcastle-Ottawa scale. All the diagnoses of depression relied on DSM. Articles were classified as having good, moderate, or poor quality using NOS scores of 9–8, 7–5, and 4–0, respectively; only those with good or moderate quality are eligible for meta-analysis.

***Table S2.* Demographic, clinical characteristics, and quality assessment of MCI studies.**

| study | Diagnostic  Tool | Sample Size  (Female%) | | Age  mean (SD) | | Education  mean (SD) | | MMSE  mean (SD) | | Medication | NOS |
| --- | --- | --- | --- | --- | --- | --- | --- | --- | --- | --- | --- |
|  |  | MCI | HCs | MCI | HCs | MCI | HCs | MCI | HCs |  |  |
| Agosta et al. ([2011](file://C:\\Users\\Administrator\\Desktop\\Supp_20240207.docx#_ENREF_1)) | Petersen (2001) | 15  (47%) | 15  (60%) | 70.40  (7.20) | 69.80  (6.00) | 9.00  (4.60) | 12.30  (3.60) | 25.80  (0.90) | 28.80  (1.50) | - | 9 |
| Barbeau et al. (2008) | Petersen (2001) | 28  (57%) | 28  (46%) | 69.30  (8.60) | 63.30  (7.20) | - | - | 27.40  (1.40) | 28.90  (1.00) | - | 5 |
| Benavides-Varela et al. (2020) | Petersen (2001) | 43  (42%) | 37  (46%) | 75.44  (47.44) | 68.89  (113.99) | 11.00  (21.67) | 12.35  (19.57) | 26.39  (2.84) | 28.73  (1.17) | - | 7 |
| Bonekamp et al. (2010) | DSM-IV | 10  (50%) | 20  (50%) | 72.70  (5.30) | 75.30  (4.80) | - | - | 26.30  (2.90) | 28.90  (1.20) | - | 5 |
| Bozzali et al. (2006) | Petersen (2001) | 14  (55%) | 20  (65%) | 70.50  (10.50) | 65.80  (6.80) | - | - | 25.80  (1.70) | 27.30  (1.20) | - | 5 |
| Bozzali et al. (2006) | Petersen (2001) | 8  (55%) | 20  (65%) | 70.50  (10.50) | 65.80  (6.80) | - | - | 24.80  (1.50) | 27.30  (1.20) | - | 5 |
| Bozzali et al. (2012) | Petersen (2001) | 23  (52%) | 14  (29%) | 71.20  (6.70) | 68.00  (8.50) | 10.30  (4.50) | 12.80  (2.90) | 24.90  (1.20) | 28.90  (1.20) | - | 6 |
| Brambati et al. (2009) | Petersen (2001) | 25  (68%) | 13  (62%) | 73.40  (6.87) | 75.00  (5.00) | 13.21  (4.49) | 14.90  (5.00) | 27.38  (1.79) | 29.10  (1.20) | - | 8 |
| Brys et al. (2009) | Petersen (2001) | 8  (88%) | 21  (71%) | 70.30  (8.30) | 65.00  (10.00) | 12.30  (3.20) | 15.00  (3.80) | 27.30  (1.90) | 29.70  (0.50) | - | 6 |
| Brys et al. (2009) | Petersen (2001) | 16  (63%) | 21  (71%) | 71.10  (6.90) | 65.00  (10.00) | 14.40  (3.60) | 15.00  (3.80) | 28.40  (1.70) | 29.70  (0.50) | - | 6 |
| Caroli et al. ([2007](file://C:\\Users\\Administrator\\Desktop\\Supp_20240207.docx#_ENREF_9)) | Petersen (2004) | 9  (44%) | 17  (53%) | 69.00  (3.00) | 69.00  (3.00) | 11.40  (5.70) | 9.80  (4.10) | 26.80  (1.80) | 27.80  (1.60) | - | 6 |
| Caroli et al. ([2007](file://C:\\Users\\Administrator\\Desktop\\Supp_20240207.docx#_ENREF_9)) | Petersen (2004) | 14  (43%) | 17  (53%) | 71.00  (8.00) | 69.00  (3.00) | 8.60  (3.60) | 9.80  (4.10) | 27.00  (2.00) | 27.80  (1.60) | - | 6 |
| Chen et al. ([2020](file://C:\\Users\\Administrator\\Desktop\\Supp_20240207.docx#_ENREF_10)) | Petersen (2004) | 20  (35%) | 29  (59%) | 71.35  (5.90) | 70.69  (5.40) | 10.88  (2.90) | 12.17  (3.20) | 27.45  (2.10) | 28.55  (1.40) | 0% | 8 |
| Chetelat et al. ([2002](file://C:\\Users\\Administrator\\Desktop\\Supp_20240207.docx#_ENREF_11)) | Petersen (2001) | 22  (55%) | 22  (55%) | 71.00  (8.00) | 66.60  (7.20) | - | - | 27.30  (1.50) | - | - | 5 |
| Clerx et al. ([2013](file://C:\\Users\\Administrator\\Desktop\\Supp_20240207.docx#_ENREF_12)) | Petersen (2001) | 18  (0%) | 18  (0%) | 65.11  (4.50) | 64.56  (3.40) | - | - | 27.61  (2.30) | 28.89  (0.90) | - | 7 |
| Defrancesco et al. ([2014](file://C:\\Users\\Administrator\\Desktop\\Supp_20240207.docx#_ENREF_14)) | Petersen (2004) | 13  (69%) | 28  (57%) | 73.30  (6.70) | 72.20  (7.10) | 10.30  (4.50) | 9.50  (3.70) | 25.20  (1.70) | 28.60  (1.20) | 0% | 7 |
| Defrancesco et al. ([2014](file://C:\\Users\\Administrator\\Desktop\\Supp_20240207.docx#_ENREF_14)) | Petersen (2004) | 14  (57%) | 28  (57%) | 72.80  (7.80) | 72.20  (7.10) | 9.60  (2.80) | 9.50  (3.70) | 27.50  (1.80) | 28.60  (1.20) | 0% | 7 |
| Derflinger et al. ([2011](file://C:\\Users\\Administrator\\Desktop\\Supp_20240207.docx#_ENREF_15)) | Petersen (2001) | 24  (54%) | 30  (67%) | 69.00  (9.00) | 67.00  (8.70) | 10.40  (2.00) | 10.60  (1.70) | 26.80  (1.70) | - | - | 7 |
| Dos Santos et al. ([2011](file://C:\\Users\\Administrator\\Desktop\\Supp_20240207.docx#_ENREF_16)) | NIA-AA | 60  (57%) | 32  (53%) | 70.33  (6.32) | 68.69  (7.36) | 9.40  (1.59) | 10.15  (2.04) | 26.38  (1.78) | 29.22  (0.79) | - | 6 |
| Eustache et al. ([2016](file://C:\\Users\\Administrator\\Desktop\\Supp_20240207.docx#_ENREF_18)) | NIA-AA | 14  (50%) | 14  (57%) | 71.10  (4.70) | 70.90  (4.60) | 11.00  (2.90) | 11.90  (2.80) | 25.80  (1.50) | 28.40  (0.80) | - | 7 |
| Ford et al. ([2014](file://C:\\Users\\Administrator\\Desktop\\Supp_20240207.docx#_ENREF_19)) | Petersen (2001) | 65  (54%) | 55  (47%) | 70.00  (-) | 75.00  (-) | 11.20  (3.20) | 11.70  (2.90) | 28.00  (-) | 29.00  (-) | - | 6 |
| Gold et al. ([2010](file://C:\\Users\\Administrator\\Desktop\\Supp_20240207.docx#_ENREF_21)) | Petersen (2001) | 12  (42%) | 14  (50%) | 77.90  (6.30) | 77.00  (4.40) | 15.30  (1.90) | 16.40  (2.70) | - | - | - | 8 |
| Gupta et al. ([2019](file://C:\\Users\\Administrator\\Desktop\\Supp_20240207.docx#_ENREF_22)) | NIA-AA | 39  (36%) | 171  (51%) | 73.24  (7.44) | 71.66  (5.43) | 8.20  (5.19) | 9.16  (5.54) | - | - | 0% | 5 |
| Haller et al. ([2014](file://C:\\Users\\Administrator\\Desktop\\Supp_20240207.docx#_ENREF_23)) | Petersen (2004) | 17  (41%) | 17  (65%) | 70.70  (4.60) | 68.30  (2.80) | 10.20  (-) | 10.20  (-) | 28.30  (1.20) | 29.20  (1.10) | - | 9 |
| Hamalainen et al. ([2007](file://C:\\Users\\Administrator\\Desktop\\Supp_20240207.docx#_ENREF_24)) | Petersen (2001) | 14  (71%) | 21  (81%) | 72.40  (7.30) | 71.20  (4.90) | 8.10  (2.60) | 7.90  (2.90) | 25.60  (3.10) | 27.70  (2.00) | 0% | 8 |
| Hamalainen et al. ([2007](file://C:\\Users\\Administrator\\Desktop\\Supp_20240207.docx#_ENREF_25)) | Petersen (2001) | 56  (73%) | 22  (50%) | 72.56  (4.09) | 72.90  (4.50) | 6.60  (1.58) | 6.80  (1.70) | 23.68  (2.25) | 26.90  (1.80) | 0% | 6 |
| Han et al. ([2021](file://C:\\Users\\Administrator\\Desktop\\Supp_20240207.docx#_ENREF_26)) | Petersen (2004) | 165  (64%) | 71  (-) | 73.50  (7.36) | 70.40  (3.90) | 8.74  (5.37) | - | 22.40  (6.10) | - | - | 7 |
| Han et al. ([2012](file://C:\\Users\\Administrator\\Desktop\\Supp_20240207.docx#_ENREF_27)) | Petersen (2001) | 17  (59%) | 18  (61%) | 69.70  (7.60) | 66.50  (6.20) | 8.80  (4.00) | 8.40  (5.60) | 25.20  (3.50) | 29.20  (0.70) | 0% | 7 |
| Hirata et al. ([2005](file://C:\\Users\\Administrator\\Desktop\\Supp_20240207.docx#_ENREF_30)) | Petersen (2001) | 30  (48%) | 41  (52%) | 70.60  (8.40) | 70.10  (7.70) | - | - | 26.00  (1.50) | 28.70  (1.50) | - | 6 |
| Hong et al. ([2015](file://C:\\Users\\Administrator\\Desktop\\Supp_20240207.docx#_ENREF_31)) | Petersen (2001) | 29  (68%) | 28  (68%) | 70.50  (5.17) | 70.60  (6.48) | 8.60  (4.36) | 8.80  (6.16) | 25.50  (2.81) | 28.70  (1.36) | - | 9 |
| Hoppstadter et al. ([2013](file://C:\\Users\\Administrator\\Desktop\\Supp_20240207.docx#_ENREF_32)) | Petersen (2004) | 14  (29%) | 10  (60%) | 68.00  (4.00) | 67.80  (4.69) | 11.30  (2.50) | 12.90  (3.80) | 27.85  (1.29) | 28.88  (1.05) | - | 7 |
| Jauhiainen et al. ([2008](file://C:\\Users\\Administrator\\Desktop\\Supp_20240207.docx#_ENREF_34)) | Petersen (2004) | 7  (43%) | 13  (85%) | 74.70  (1.90) | 74.40  (1.20) | 7.90  (1.00) | 8.80  (0.70) | 25.40  (1.00) | 27.90  (0.70) | - | 7 |
| Kang et al. ([2019](file://C:\\Users\\Administrator\\Desktop\\Supp_20240207.docx#_ENREF_35)) | Petersen (2001) | 30  (47%) | 37  (60%) | 76.90  (4.30) | 73.90  (2.00) | 9.00  (4.80) | 10.80  (4.00) | 21.70  (4.30) | 27.10  (1.70) | 0% | 6 |
| Kang et al. ([2019](file://C:\\Users\\Administrator\\Desktop\\Supp_20240207.docx#_ENREF_35)) | Petersen (2001) | 35  (54%) | 37  (60%) | 77.30  (4.10) | 73.90  (2.00) | 9.60  (4.30) | 10.80  (4.00) | 21.70  (4.50) | 27.10  (1.70) | 0% | 6 |
| Kim et al. ([2020](file://C:\\Users\\Administrator\\Desktop\\Supp_20240207.docx#_ENREF_36)) | DSM-IV | 10  (70%) | 9  (67%) | 73.10  (7.90) | 70.70  (3.50) | - | - | 16.50  (4.90) | 28.60  (1.10) | 0% | 5 |
| Kunst et al. ([2019](file://C:\\Users\\Administrator\\Desktop\\Supp_20240207.docx#_ENREF_38)) | Petersen (2001) | 27  (63%) | 58  (69%) | 69.80  (6.90) | 67.50  (7.30) | 14.30  (3.00) | 15.40  (2.50) | 27.10  (1.30) | 28.50  (1.20) | - | 6 |
| Lee et al. ([2010](file://C:\\Users\\Administrator\\Desktop\\Supp_20240207.docx#_ENREF_39)) | Petersen (2001) | 78  (59%) | 21  (-) | 70.50  (8.00) | 70.70  (2.70) | 9.40  (4.90) | - | 25.10  (2.40) | - | - | 7 |
| Liu et al. ([2022](file://C:\\Users\\Administrator\\Desktop\\Supp_20240207.docx#_ENREF_40)) | Petersen (2001) | 114  (60%) | 101  (64%) | 72.35  (5.23) | 71.69  (4.95) | 10.78  (3.71) | 10.24  (2.73) | 24.11  (1.01) | 28.31  (0.97) | - | 7 |
| Mitolo et al. ([2013](file://C:\\Users\\Administrator\\Desktop\\Supp_20240207.docx#_ENREF_42)) | Petersen (2001) | 20  (50%) | 14  (71%) | 74.75  (6.93) | 68.64  (4.53) | 7.85  (4.39) | 8.57  (4.88) | 25.80  (3.35) | 29.57  (0.75) | - | 7 |
| Pa et al. ([2009](file://C:\\Users\\Administrator\\Desktop\\Supp_20240207.docx#_ENREF_44)) | Petersen (2004) | 26  (50%) | 36  (64%) | 68.00  (6.60) | 64.80  (8.20) | 17.50  (1.70) | 17.00  (2.00) | 28.70  (1.20) | 29.80  (0.60) | - | 6 |
| Pennanen et al. ([2005](file://C:\\Users\\Administrator\\Desktop\\Supp_20240207.docx#_ENREF_45)) | Petersen (2001) | 51  (67%) | 32  (59%) | 72.00  (5.00) | 74.00  (4.00) | 7.00  (2.00) | 7.00  (2.00) | 24.00  (2.00) | 27.00  (2.00) | - | 7 |
| Rami et al. ([2009](file://C:\\Users\\Administrator\\Desktop\\Supp_20240207.docx#_ENREF_46)) | Petersen (2001) | 14  (69%) | 27  (63%) | 72.90  (4.80) | 74.30  (5.30) | 7.40  (4.20) | 9.40  (5.20) | 26.00  (2.00) | 27.40  (1.00) | - | 6 |
| Rami et al. ([2012](file://C:\\Users\\Administrator\\Desktop\\Supp_20240207.docx#_ENREF_47)) | Petersen (2001) | 14  (-) | 24  (-) | 71.60  (5.30) | 71.40  (6.60) | 8.50  (5.00) | 9.00  (4.70) | 24.80  (1.60) | 28.10  (1.40) | - | 6 |
| Remillard-Pelchat et al. ([2022](file://C:\\Users\\Administrator\\Desktop\\Supp_20240207.docx#_ENREF_48)) | Petersen (2004) | 17  (29%) | 41  (39%) | 67.90  (4.40) | 63.20  (8.20) | 12.10  (3.80) | 14.60  (4.10) | - | - | - | 6 |
| Saykin et al. ([2006](file://C:\\Users\\Administrator\\Desktop\\Supp_20240207.docx#_ENREF_51)) | Petersen (2004) | 40  (43%) | 40  (70%) | 72.90  (7.10) | 71.00  (5.10) | 16.30  (3.30) | 16.60  (2.70) | 27.20  (2.20) | 29.10  (1.00) | 3% | 8 |
| Schmidt-Wilcke et al. ([2009](file://C:\\Users\\Administrator\\Desktop\\Supp_20240207.docx#_ENREF_52)) | Petersen (2001) | 18  (44%) | 18  (44%) | 65.70  (7.20) | 63.00  (10.70) | 13.00  (3.60) | 12.60  (3.30) | - | - | - | 8 |
| Serra et al. ([2013](file://C:\\Users\\Administrator\\Desktop\\Supp_20240207.docx#_ENREF_53)) | Petersen (2004) | 15  (27%) | 28  (37%) | 70.90  (9.00) | 63.40  (8.90) | 11.30  (4.40) | 13.10  (3.50) | 25.40  (1.70) | 28.40  (1.70) | - | 6 |
| Shiino et al. ([2006](file://C:\\Users\\Administrator\\Desktop\\Supp_20240207.docx#_ENREF_54)) | Petersen (2004) | 20  (50%) | 88  (55%) | 67.70  (9.00) | 68.70  (8.70) | - | - | 26.80  (1.88) | 29.09  (1.47) | - | 6 |
| Trivedi et al. ([2006](file://C:\\Users\\Administrator\\Desktop\\Supp_20240207.docx#_ENREF_56)) | Petersen (2004) | 15  (40%) | 15  (40%) | 73.30  (6.72) | 73.60  (7.10) | 16.30  (2.81) | 16.70  (2.50) | 27.80  (1.80) | 29.70  (0.50) | - | 8 |
| van de Mortel et al. ([2021](file://C:\\Users\\Administrator\\Desktop\\Supp_20240207.docx#_ENREF_58)) | NIA-AA | 295  (43%) | 351  (54%) | 71.00  (7.47) | 75.00  (5.79) | 15.90  (2.64) | 16.30  (2.70) | 28.30  (1.55) | 29.10  (1.13) | 0% | 6 |
| Venneri et al. ([2011](file://C:\\Users\\Administrator\\Desktop\\Supp_20240207.docx#_ENREF_59)) | Petersen (2001) | 25  (48%) | 25  (60%) | 70.52  (6.44) | 70.29  (6.49) | 8.96  (4.41) | 9.32  (4.46) | 28.24  (1.23) | 28.68  (1.52) | - | 7 |
| Wang et al. ([2012](file://C:\\Users\\Administrator\\Desktop\\Supp_20240207.docx#_ENREF_60)) | Petersen (2001) | 40  (40%) | 30  (37%) | 77.30  (6.60) | 76.10  (7.20) | 11.40  (4.30) | 13.50  (2.60) | - | - | - | 6 |
| Xie et al. ([2012](file://C:\\Users\\Administrator\\Desktop\\Supp_20240207.docx#_ENREF_61)) | Petersen (2004) | 17  (65%) | 25  (48%) | 75.12  (6.62) | 74.28  (8.25) | 13.47  (2.07) | 15.32  (2.87) | 27.29  (1.83) | 28.92  (1.22) | 48% | 6 |
| You et al. ([2021](file://C:\\Users\\Administrator\\Desktop\\Supp_20240207.docx#_ENREF_62)) | NIA-AA | 60  (55%) | 30  (57%) | 67.32  (9.44) | 64.67  (8.01) | 9.00  (-) | 10.00  (-) | 24.00  (-) | 28.00  (-) | 0% | 7 |
| Zhao et al. ([2015](file://C:\\Users\\Administrator\\Desktop\\Supp_20240207.docx#_ENREF_64)) | Petersen (2001) | 34  (59%) | 34  (47%) | 68.00  (7.60) | 66.90  (6.70) | 10.80  (3.30) | 11.50  (3.90) | 25.50  (1.60) | 29.20  (0.90) | - | 7 |
| Mean/Summary |  | 1878  (52%) | 2046  (52%) | 71.57 | 70.82 | 11.30 | 12.40 | 25.83 | 28.65 |  |  |

MCI: mild cognitive impairment; NIA-AA: National Institute on Aging and Alzheimer’s Association; DSM: Diagnostic and Statistical Manual of Mental Disorders; MMSE, Mini Mental State Examination; NOS, Newcastle-Ottawa scale. All the diagnoses of MCI relied on reliable clinical criteria. The criteria proposed by Petersen (2001) include (i) memory complaint, preferably corroborated by an informant; (ii) impaired memory function for age and education; (iii) preserved general cognitive function; (iv) intact activities of daily living; and (v) not demented. The criteria proposed by Petersen (2004) include (i) memory complaint usually corroborated by an informant; (ii) objective memory impairment for age; (iii) essentially preserved general cognitive function; (iv) largely intact functional activities; and (v) not demented. Articles were classified as having good, moderate, or poor quality using NOS scores of 9–8, 7–5, and 4–0, respectively; only those with good or moderate quality are eligible for meta-analysis.

***Table S3.* Results of meta-analysis, heterogeneity assessment and publication bias of GM atrophy in LLD and MCI.**

| Cluster | | Peak | | | *I^2^* | Egger's *p* |
| --- | --- | --- | --- | --- | --- | --- |
| Description | Voxels | MNI (x,y,z) | SDM-*Z* | *p* |  |  |
| *LLD < HC* |  |  |  |  |  |  |
| R. gyrus rectus | 1215 | 2,22,-18 | -3.237 | 0.0006 | 4.70 | 0.669 |
| L.middle temporal gyrus | 154 | -50,-66,12 | -2.503 | 0.0061 | 13.55 | 0.903 |
| L. inferior frontal gyrus, orbital part | 96 | -38,32,-12 | -2.466 | 0.0068 | 6.88 | 0.801 |
| R. anterior thalamic projections | 78 | 46,32,6 | -2.29 | 0.0110 | 3.31 | 0.558 |
| L. anterior cingulate/ paracingulate gyri | 63 | 0,42,8 | -2.172 | 0.0149 | 6.12 | 0.799 |
| R. insula | 36 | 42,10,-4 | -2.055 | 0.0199 | 1.60 | 0.513 |
| R. median cingulate/ paracingulate gyri | 32 | 12,-40,44 | -2.143 | 0.0161 | 14.11 | 0.339 |
| L. insula | 24 | -30,16,2 | -1.898 | 0.0288 | 35.04 | 0.493 |
| L. middle temporal gyrus | 24 | -60,-46,2 | -1.908 | 0.0282 | 16.20 | 0.928 |
| *MCI < HC* |  |  |  |  |  |  |
| R. parahippocampal gyrus | 12069 | 24,-2,-28 | -5.342 | <0.001 | 49.69 | 0.181 |
| L. hippocampus | 6143 | -26,-8,-14 | -5.831 | <0.001 | 5.96 | 0.251 |
| L. precuneus | 2325 | 0,-44,38 | -3.693 | 0.001 | 0.21 | 0.967 |
| L. angular gyrus | 656 | -50,-62,28 | -2.831 | 0.0023 | 51.24 | 0.312 |

R., right; L., left; MNI, Montreal Neurological Institute; SDM, signed differential mapping; uncorrected *p*<0.05, and voxel extent ≥10.

***Table S4.* Shared abnormal regions in GM between LLD and MCI.**

| Cluster | | Peak | *I^2^* | |
| --- | --- | --- | --- | --- |
| Region | Voxels | MNI (x,y,z) | MCI < HC | LLD < HC |
| R.median cingulate/  paracingulate gyri | 16 | 4,-30,50 | 3.83 | 27.75 |
| R. insula | 10 | 34,16,2 | 3.09 | 6.59 |

R., right; L., left; MNI, Montreal Neurological Institute; threshold with p=0.0025, peak height threshold with *p*=0.00025, voxel extent ≥10.

***Table S5.* Discrepant abnormal regions in GM between LLD and MCI.**

| Cluster | | Peak | | | *I^2^* | Egger's *p* |
| --- | --- | --- | --- | --- | --- | --- |
| Region | Voxels | MNI (x,y,z) | SDM-*Z* | *p* |  |  |
| L. hippocampus | 179 | -26,-14,-18 | -2.251 | 0.0122 | 9.78 | 0.492 |
| R. parahippocampal gyrus | 86 | 26,0,-32 | -2.017 | 0.0219 | 24.22 | 0.574 |

R., right; L., left; MNI, Montreal Neurological Institute; SDM, signed differential mapping; uncorrected *p*<0.05, and voxel extent ≥1.


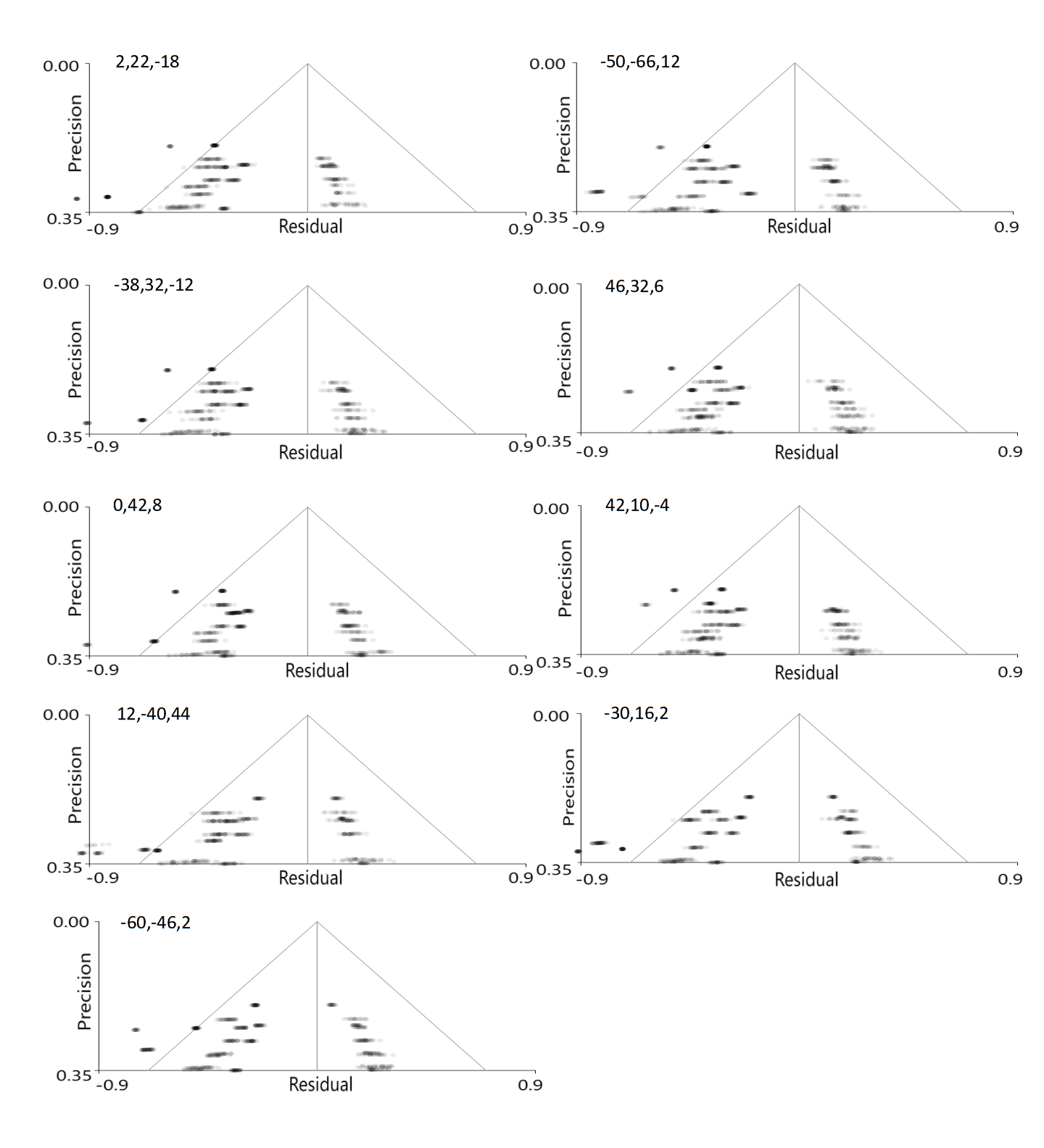


***Figure S1.* Funnel plot analysis for significant coordinates in comparison between LLDs and HCs.**


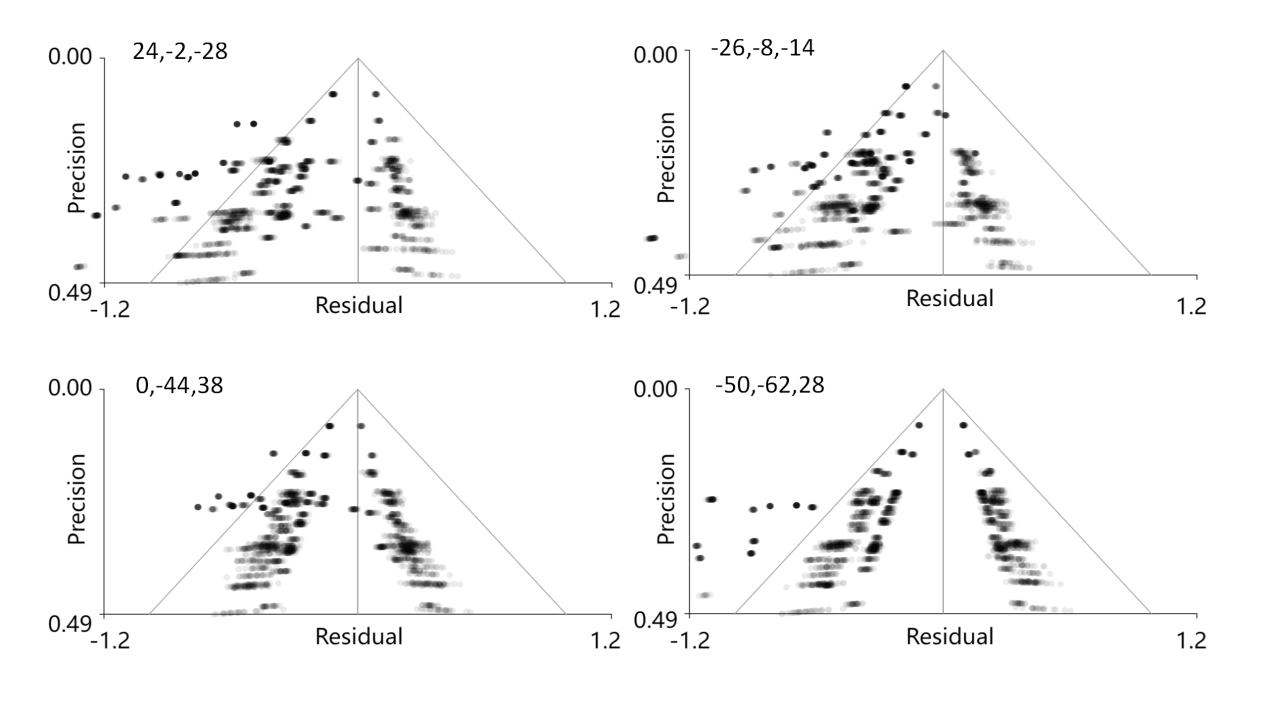


***Figure S2.* Funnel plot analysis for significant coordinates in comparison between MCIs and HCs.**


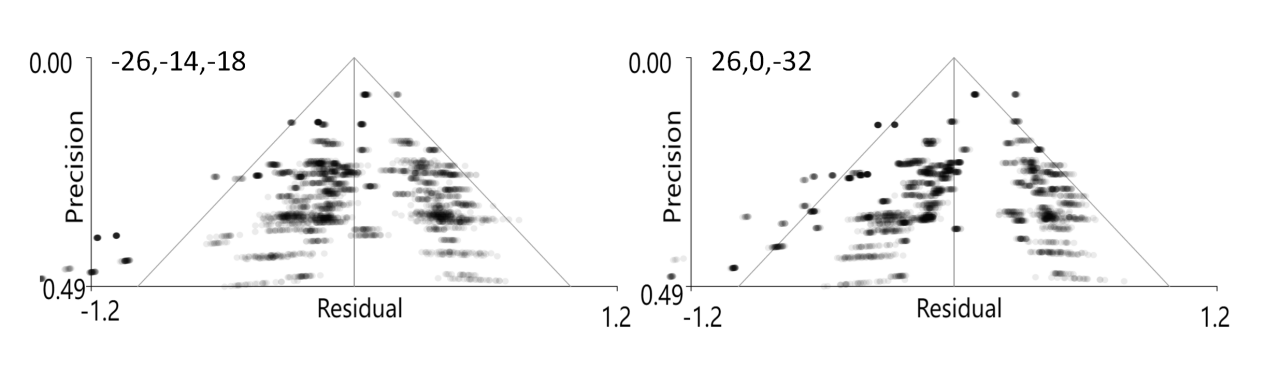


***Figure S3.*** **Funnel plot analysis for significant coordinates in conjunction analysis (shared abnormality between LLD and MCI).**

**Included studies on late life depression**

Colloby, S. J., Firbank, M. J., Vasudev, A., Parry, S. W., Thomas, A. J., & O'Brien, J. T. (2011). Cortical thickness and VBM-DARTEL in late-life depression. J Affect Disord, 133(1-2), 158-164. doi:10.1016/j.jad.2011.04.010

Egger, K., Schocke, M., Weiss, E., Auffinger, S., Esterhammer, R., Goebel, G., . . . Marksteiner, J. (2008). Pattern of brain atrophy in elderly patients with depression revealed by voxel-based morphometry. Psychiatry Res, 164(3), 237-244. doi:10.1016/j.pscychresns.2007.12.018

Harada, K., Matsuo, K., Nakashima, M., Hobara, T., Higuchi, N., Higuchi, F., . . . Watanabe, Y. (2016). Disrupted orbitomedial prefrontal limbic network in individuals with later-life depression. J Affect Disord, 204, 112-119. doi:10.1016/j.jad.2016.06.031

Hwang, J. P., Lee, T. W., Tsai, S. J., Chen, T. J., Yang, C. H., Lirng, J. F., & Tsai, C. F. (2010). Cortical and subcortical abnormalities in late-onset depression with history of suicide attempts investigated with MRI and voxel-based morphometry. J Geriatr Psychiatry Neurol, 23(3), 171-184. doi:10.1177/0891988710363713

Koolschijn, P. C., van Haren, N. E., Schnack, H. G., Janssen, J., Hulshoff Pol, H. E., & Kahn, R. S. (2010). Cortical thickness and voxel-based morphometry in depressed elderly. Eur Neuropsychopharmacol, 20(6), 398-404. doi:10.1016/j.euroneuro.2010.02.010

Marano, C. M., Workman, C. I., Lyman, C. H., Munro, C. A., Kraut, M. A., & Smith, G. S. (2015). Structural imaging in late-life depression: association with mood and cognitive responses to antidepressant treatment. Am J Geriatr Psychiatry, 23(1), 4-12. doi:10.1016/j.jagp.2013.10.001

Oudega, M. L., van Exel, E., Stek, M. L., Wattjes, M. P., van der Flier, W. M., Comijs, H. C., . . . van den Heuvel, O. A. (2014). The structure of the geriatric depressed brain and response to electroconvulsive therapy. Psychiatry Res, 222(1-2), 1-9. doi:10.1016/j.pscychresns.2014.03.002

Ribeiz, S. R., Duran, F., Oliveira, M. C., Bezerra, D., Castro, C. C., Steffens, D. C., . . . Bottino, C. M. (2013). Structural brain changes as biomarkers and outcome predictors in patients with late-life depression: a cross-sectional and prospective study. PLoS One, 8(11), e80049. doi:10.1371/journal.pone.0080049

Ries, M. L., Wichmann, A., Bendlin, B. B., & Johnson, S. C. (2009). Posterior Cingulate and Lateral Parietal Gray Matter Volume in Older Adults with Depressive Symptoms. Brain Imaging Behav, 3(3), 233-239. doi:10.1007/s11682-009-9065-4

Sin, E. L. L., Liu, H. L., Lee, S. H., Huang, C. M., Wai, Y. Y., Chen, Y. L., . . . Lee, T. M. C. (2018). The relationships between brain structural changes and perceived loneliness in older adults suffering from late-life depression. Int J Geriatr Psychiatry, 33(4), 606-612. doi:10.1002/gps.4831

Tsai, C. F., Chuang, C. H., Wang, Y. P., Lin, Y. B., Tu, P. C., Liu, P. Y., . . . Lu, C. L. (2022). Differences in gut microbiota correlate with symptoms and regional brain volumes in patients with late-life depression. Front Aging Neurosci, 14, 885393. doi:10.3389/fnagi.2022.885393

Xie, C., Li, W., Chen, G., Douglas Ward, B., Franczak, M. B., Jones, J. L., . . . Goveas, J. S. (2012). The co-existence of geriatric depression and amnestic mild cognitive impairment detrimentally affect gray matter volumes: voxel-based morphometry study. Behav Brain Res, 235(2), 244-250. doi:10.1016/j.bbr.2012.08.007

Yuan, Y., Zhu, W., Zhang, Z., Bai, F., Yu, H., Shi, Y., . . . Liu, Z. (2008). Regional gray matter changes are associated with cognitive deficits in remitted geriatric depression: an optimized voxel-based morphometry study. Biol Psychiatry, 64(6), 541-544. doi:10.1016/j.biopsych.2008.04.032

**Included studies on mild cognitive impairment**

Agosta, F., Pievani, M., Sala, S., Geroldi, C., Galluzzi, S., Frisoni, G. B., & Filippi, M. (2011). White matter damage in Alzheimer disease and its relationship to gray matter atrophy. Radiology, 258(3), 853-863. doi:10.1148/radiol.10101284

Barbeau, E. J., Ranjeva, J. P., Didic, M., Confort-Gouny, S., Felician, O., Soulier, E., . . . Poncet, M. (2008). Profile of memory impairment and gray matter loss in amnestic mild cognitive impairment. Neuropsychologia, 46(4), 1009-1019. doi:10.1016/j.neuropsychologia.2007.11.019

Benavides-Varela, S., Burgio, F., Weis, L., Mitolo, M., Palmer, K., Toffano, R., . . . Semenza, C. (2020). The role of limbic structures in financial abilities of mild cognitive impairment patients. Neuroimage Clin, 26, 102222. doi:10.1016/j.nicl.2020.102222

Bonekamp, D., Yassa, M. A., Munro, C. A., Geckle, R. J., Yousem, D. M., Barker, P. B., . . . Horska, A. (2010). Gray matter in amnestic mild cognitive impairment: voxel-based morphometry. Neuroreport, 21(4), 259-263. doi:10.1097/WNR.0b013e328335642a

Bozzali, M., Filippi, M., Magnani, G., Cercignani, M., Franceschi, M., Schiatti, E., . . . Falini, A. (2006). The contribution of voxel-based morphometry in staging patients with mild cognitive impairment. Neurology, 67(3), 453-460. doi:10.1212/01.wnl.0000228243.56665.c2

Bozzali, M., Giulietti, G., Basile, B., Serra, L., Spano, B., Perri, R., . . . Cercignani, M. (2012). Damage to the cingulum contributes to Alzheimer's disease pathophysiology by deafferentation mechanism. Hum Brain Mapp, 33(6), 1295-1308. doi:10.1002/hbm.21287

Brambati, S. M., Belleville, S., Kergoat, M. J., Chayer, C., Gauthier, S., & Joubert, S. (2009). Single- and multiple-domain amnestic mild cognitive impairment: two sides of the same coin? Dement Geriatr Cogn Disord, 28(6), 541-549. doi:10.1159/000255240

Brys, M., Glodzik, L., Mosconi, L., Switalski, R., De Santi, S., Pirraglia, E., . . . de Leon, M. J. (2009). Magnetic resonance imaging improves cerebrospinal fluid biomarkers in the early detection of Alzheimer's disease. J Alzheimers Dis, 16(2), 351-362. doi:10.3233/JAD-2009-0968

Caroli, A., Testa, C., Geroldi, C., Nobili, F., Barnden, L. R., Guerra, U. P., . . . Frisoni, G. B. (2007). Cerebral perfusion correlates of conversion to Alzheimer's disease in amnestic mild cognitive impairment. J Neurol, 254(12), 1698-1707. doi:10.1007/s00415-007-0631-7

Chen, J., Yan, Y., Gu, L., Gao, L., & Zhang, Z. (2020). Electrophysiological Processes on Motor Imagery Mediate the Association Between Increased Gray Matter Volume and Cognition in Amnestic Mild Cognitive Impairment. Brain Topogr, 33(2), 255-266. doi:10.1007/s10548-019-00742-8

Chetelat, G., Desgranges, B., De La Sayette, V., Viader, F., Eustache, F., & Baron, J. C. (2002). Mapping gray matter loss with voxel-based morphometry in mild cognitive impairment. Neuroreport, 13(15), 1939-1943. doi:10.1097/00001756-200210280-00022

Clerx, L., Jacobs, H. I., Burgmans, S., Gronenschild, E. H., Uylings, H. B., Echavarri, C., . . . Aalten, P. (2013). Sensitivity of different MRI-techniques to assess gray matter atrophy patterns in Alzheimer's disease is region-specific. Curr Alzheimer Res, 10(9), 940-951. doi:10.2174/15672050113109990158

Defrancesco, M., Egger, K., Marksteiner, J., Esterhammer, R., Hinterhuber, H., Deisenhammer, E. A., & Schocke, M. (2014). Changes in white matter integrity before conversion from mild cognitive impairment to Alzheimer's disease. PLoS One, 9(8), e106062. doi:10.1371/journal.pone.0106062

Derflinger, S., Sorg, C., Gaser, C., Myers, N., Arsic, M., Kurz, A., . . . Muhlau, M. (2011). Grey-matter atrophy in Alzheimer's disease is asymmetric but not lateralized. J Alzheimers Dis, 25(2), 347-357. doi:10.3233/JAD-2011-110041

Dos Santos, V., Thomann, P. A., Wustenberg, T., Seidl, U., Essig, M., & Schroder, J. (2011). Morphological cerebral correlates of CERAD test performance in mild cognitive impairment and Alzheimer's disease. J Alzheimers Dis, 23(3), 411-420. doi:10.3233/JAD-2010-100156

Eustache, P., Nemmi, F., Saint-Aubert, L., Pariente, J., & Peran, P. (2016). Multimodal Magnetic Resonance Imaging in Alzheimer's Disease Patients at Prodromal Stage. J Alzheimers Dis, 50(4), 1035-1050. doi:10.3233/JAD-150353

Ford, A. H., Almeida, O. P., Flicker, L., Garrido, G. J., Greenop, K. R., Foster, J. K., . . . Lautenschlager, N. T. (2014). Grey matter changes associated with deficit awareness in mild cognitive impairment: a voxel-based morphometry study. J Alzheimers Dis, 42(4), 1251-1259. doi:10.3233/JAD-132678

Gold, B. T., Jiang, Y., Jicha, G. A., & Smith, C. D. (2010). Functional response in ventral temporal cortex differentiates mild cognitive impairment from normal aging. Hum Brain Mapp, 31(8), 1249-1259. doi:10.1002/hbm.20932

Gupta, Y., Lee, K. H., Choi, K. Y., Lee, J. J., Kim, B. C., Kwon, G. R., . . . Alzheimer's Disease Neuroimaging, I. (2019). Early diagnosis of Alzheimer's disease using combined features from voxel-based morphometry and cortical, subcortical, and hippocampus regions of MRI T1 brain images. PLoS One, 14(10), e0222446. doi:10.1371/journal.pone.0222446

Haller, S., Montandon, M. L., Rodriguez, C., Moser, D., Toma, S., Hofmeister, J., . . . Giannakopoulos, P. (2014). Acute caffeine administration effect on brain activation patterns in mild cognitive impairment. J Alzheimers Dis, 41(1), 101-112. doi:10.3233/JAD-132360

Hamalainen, A., Pihlajamaki, M., Tanila, H., Hanninen, T., Niskanen, E., Tervo, S., . . . Soininen, H. (2007). Increased fMRI responses during encoding in mild cognitive impairment. Neurobiol Aging, 28(12), 1889-1903. doi:10.1016/j.neurobiolaging.2006.08.008

Hamalainen, A., Tervo, S., Grau-Olivares, M., Niskanen, E., Pennanen, C., Huuskonen, J., . . . Soininen, H. (2007). Voxel-based morphometry to detect brain atrophy in progressive mild cognitive impairment. Neuroimage, 37(4), 1122-1131. doi:10.1016/j.neuroimage.2007.06.016

Han, S. H., Pyun, J. M., Yeo, S., Kang, D. W., Jeong, H. T., Kang, S. W., . . . Youn, Y. C. (2021). Differences between memory encoding and retrieval failure in mild cognitive impairment: results from quantitative electroencephalography and magnetic resonance volumetry. Alzheimers Res Ther, 13(1), 3. doi:10.1186/s13195-020-00739-7

Han, Y., Lui, S., Kuang, W., Lang, Q., Zou, L., & Jia, J. (2012). Anatomical and functional deficits in patients with amnestic mild cognitive impairment. PLoS One, 7(2), e28664. doi:10.1371/journal.pone.0028664

Hirata, Y., Matsuda, H., Nemoto, K., Ohnishi, T., Hirao, K., Yamashita, F., . . . Samejima, H. (2005). Voxel-based morphometry to discriminate early Alzheimer's disease from controls. Neurosci Lett, 382(3), 269-274. doi:10.1016/j.neulet.2005.03.038

Hong, Y. J., Yoon, B., Shim, Y. S., Ahn, K. J., Yang, D. W., & Lee, J. H. (2015). Gray and White Matter Degenerations in Subjective Memory Impairment: Comparisons with Normal Controls and Mild Cognitive Impairment. J Korean Med Sci, 30(11), 1652-1658. doi:10.3346/jkms.2015.30.11.1652

Hoppstadter, M., King, A. V., Frolich, L., Wessa, M., Flor, H., & Meyer, P. (2013). A combined electrophysiological and morphological examination of episodic memory decline in amnestic mild cognitive impairment. Front Aging Neurosci, 5, 51. doi:10.3389/fnagi.2013.00051

Jauhiainen, A. M., Kangasmaa, T., Rusanen, M., Niskanen, E., Tervo, S., Kivipelto, M., . . . Soininen, H. (2008). Differential hypometabolism patterns according to mild cognitive impairment subtypes. Dement Geriatr Cogn Disord, 26(6), 490-498. doi:10.1159/000167880

Kang, D. W., Lim, H. K., Joo, S. H., Lee, N. R., & Lee, C. U. (2019). Differential Associations Between Volumes of Atrophic Cortical Brain Regions and Memory Performances in Early and Late Mild Cognitive Impairment. Front Aging Neurosci, 11, 245. doi:10.3389/fnagi.2019.00245

Kim, G. W., Park, S. E., Park, K., & Jeong, G. W. (2020). White Matter Connectivity and Gray Matter Volume Changes Following Donepezil Treatment in Patients With Mild Cognitive Impairment: A Preliminary Study Using Probabilistic Tractography. Front Aging Neurosci, 12, 604940. doi:10.3389/fnagi.2020.604940

Kunst, J., Marecek, R., Klobusiakova, P., Balazova, Z., Anderkova, L., Nemcova-Elfmarkova, N., & Rektorova, I. (2019). Patterns of Grey Matter Atrophy at Different Stages of Parkinson's and Alzheimer's Diseases and Relation to Cognition. Brain Topogr, 32(1), 142-160. doi:10.1007/s10548-018-0675-2

Lee, J. E., Park, H. J., Song, S. K., Sohn, Y. H., Lee, J. D., & Lee, P. H. (2010). Neuroanatomic basis of amnestic MCI differs in patients with and without Parkinson disease. Neurology, 75(22), 2009-2016. doi:10.1212/WNL.0b013e3181ff96bf

Liu, L., Wang, T., Du, X., Zhang, X., Xue, C., Ma, Y., & Wang, D. (2022). Concurrent Structural and Functional Patterns in Patients With Amnestic Mild Cognitive Impairment. Front Aging Neurosci, 14, 838161. doi:10.3389/fnagi.2022.838161

Mitolo, M., Gardini, S., Fasano, F., Crisi, G., Pelosi, A., Pazzaglia, F., & Caffarra, P. (2013). Visuospatial memory and neuroimaging correlates in mild cognitive impairment. J Alzheimers Dis, 35(1), 75-90. doi:10.3233/JAD-121288

Pa, J., Boxer, A., Chao, L. L., Gazzaley, A., Freeman, K., Kramer, J., . . . Johnson, J. K. (2009). Clinical-neuroimaging characteristics of dysexecutive mild cognitive impairment. Ann Neurol, 65(4), 414-423. doi:10.1002/ana.21591

Pennanen, C., Testa, C., Laakso, M. P., Hallikainen, M., Helkala, E. L., Hanninen, T., . . . Soininen, H. (2005). A voxel based morphometry study on mild cognitive impairment. J Neurol Neurosurg Psychiatry, 76(1), 11-14. doi:10.1136/jnnp.2004.035600

Rami, L., Gomez-Anson, B., Monte, G. C., Bosch, B., Sanchez-Valle, R., & Molinuevo, J. L. (2009). Voxel based morphometry features and follow-up of amnestic patients at high risk for Alzheimer's disease conversion. Int J Geriatr Psychiatry, 24(8), 875-884. doi:10.1002/gps.2216

Rami, L., Sole-Padulles, C., Fortea, J., Bosch, B., Llado, A., Antonell, A., . . . Molinuevo, J. L. (2012). Applying the new research diagnostic criteria: MRI findings and neuropsychological correlations of prodromal AD. Int J Geriatr Psychiatry, 27(2), 127-134. doi:10.1002/gps.2696

Remillard-Pelchat, D., Rahayel, S., Gaubert, M., Postuma, R. B., Montplaisir, J., Pelletier, A., . . . Gagnon, J. F. (2022). Comprehensive Analysis of Brain Volume in REM Sleep Behavior Disorder with Mild Cognitive Impairment. J Parkinsons Dis, 12(1), 229-241. doi:10.3233/JPD-212691

Saykin, A. J., Wishart, H. A., Rabin, L. A., Santulli, R. B., Flashman, L. A., West, J. D., . . . Mamourian, A. C. (2006). Older adults with cognitive complaints show brain atrophy similar to that of amnestic MCI. Neurology, 67(5), 834-842. doi:10.1212/01.wnl.0000234032.77541.a2

Schmidt-Wilcke, T., Poljansky, S., Hierlmeier, S., Hausner, J., & Ibach, B. (2009). Memory performance correlates with gray matter density in the ento-/perirhinal cortex and posterior hippocampus in patients with mild cognitive impairment and healthy controls--a voxel based morphometry study. Neuroimage, 47(4), 1914-1920. doi:10.1016/j.neuroimage.2009.04.092

Serra, L., Giulietti, G., Cercignani, M., Spano, B., Torso, M., Castelli, D., . . . Bozzali, M. (2013). Mild cognitive impairment: same identity for different entities. J Alzheimers Dis, 33(4), 1157-1165. doi:10.3233/JAD-2012-121663

Shiino, A., Watanabe, T., Maeda, K., Kotani, E., Akiguchi, I., & Matsuda, M. (2006). Four subgroups of Alzheimer's disease based on patterns of atrophy using VBM and a unique pattern for early onset disease. Neuroimage, 33(1), 17-26. doi:10.1016/j.neuroimage.2006.06.010

Trivedi, M. A., Wichmann, A. K., Torgerson, B. M., Ward, M. A., Schmitz, T. W., Ries, M. L., . . . Johnson, S. C. (2006). Structural MRI discriminates individuals with Mild Cognitive Impairment from age-matched controls: a combined neuropsychological and voxel based morphometry study. Alzheimers Dement, 2(4), 296-302. doi:10.1016/j.jalz.2006.06.001

van de Mortel, L. A., Thomas, R. M., van Wingen, G. A., & Alzheimer's Disease Neuroimaging, I. (2021). Grey Matter Loss at Different Stages of Cognitive Decline: A Role for the Thalamus in Developing Alzheimer's Disease. J Alzheimers Dis, 83(2), 705-720. doi:10.3233/JAD-210173

Venneri, A., Gorgoglione, G., Toraci, C., Nocetti, L., Panzetti, P., & Nichelli, P. (2011). Combining neuropsychological and structural neuroimaging indicators of conversion to Alzheimer's disease in amnestic mild cognitive impairment. Curr Alzheimer Res, 8(7), 789-797. doi:10.2174/156720511797633160

Wang, P. N., Chou, K. H., Lirng, J. F., Lin, K. N., Chen, W. T., & Lin, C. P. (2012). Multiple diffusivities define white matter degeneration in amnestic mild cognitive impairment and Alzheimer's disease. J Alzheimers Dis, 30(2), 423-437. doi:10.3233/JAD-2012-111304

Xie, C., Li, W., Chen, G., Douglas Ward, B., Franczak, M. B., Jones, J. L., . . . Goveas, J. S. (2012). The co-existence of geriatric depression and amnestic mild cognitive impairment detrimentally affect gray matter volumes: voxel-based morphometry study. Behav Brain Res, 235(2), 244-250. doi:10.1016/j.bbr.2012.08.007

You, M., Zhou, X., Yin, W., Wan, K., Zhang, W., Li, C., . . . Sun, Z. (2021). The Influence of MTHFR Polymorphism on Gray Matter Volume in Patients With Amnestic Mild Cognitive Impairment. Front Neurosci, 15, 778123. doi:10.3389/fnins.2021.778123

Zhao, Z. L., Fan, F. M., Lu, J., Li, H. J., Jia, L. F., Han, Y., & Li, K. C. (2015). Changes of gray matter volume and amplitude of low-frequency oscillations in amnestic MCI: An integrative multi-modal MRI study. Acta Radiol, 56(5), 614-621. doi:10.1177/0284185114533329

**References**

Glasser, M. F., Coalson, T. S., Robinson, E. C., Hacker, C. D., Harwell, J., Yacoub, E., . . . Van Essen, D. C. (2016). A multi-modal parcellation of human cerebral cortex. *Nature, 536*(7615), 171-178. doi:10.1038/nature18933

Hawrylycz, M., Miller, J. A., Menon, V., Feng, D., Dolbeare, T., Guillozet-Bongaarts, A. L., . . . Lein, E. (2015). Canonical genetic signatures of the adult human brain. *Nat Neurosci, 18*(12), 1832-1844. doi:10.1038/nn.4171

Petersen, R. C. (2004). Mild cognitive impairment as a diagnostic entity. *J Intern Med, 256*(3), 183-194. doi:10.1111/j.1365-2796.2004.01388.x

Petersen, R. C., Doody, R., Kurz, A., Mohs, R. C., Morris, J. C., Rabins, P. V., . . . Winblad, B. (2001). Current concepts in mild cognitive impairment. *Arch Neurol, 58*(12), 1985-1992. doi:10.1001/archneur.58.12.1985
